# Deep‐Learning‐Based Approaches for Rational Design of Stapled Peptides With High Antimicrobial Activity and Stability

**DOI:** 10.1111/1751-7915.70121

**Published:** 2025-03-05

**Authors:** Ruole Chen, Yuhao You, Yanchao Liu, Xin Sun, Tianyue Ma, Xingzhen Lao, Heng Zheng

**Affiliations:** ^1^ School of Life Science and Technology China Pharmaceutical University Nanjing Jiangsu China

**Keywords:** antimicrobial activity, antimicrobial peptides, deep learning, haemolytic activity, serum stability, stapled peptides

## Abstract

Antimicrobial peptides (AMPs) face stability and toxicity challenges in clinical use. Stapled modification enhances their stability and effectiveness, but its application in peptide design is rarely reported. This study built ten prediction models for stapled AMPs using deep and machine learning, tested their accuracy with an independent data set and wet lab experiments, and characterised stapled loop structures using structural, sequence and amino acid descriptors. AlphaFold improved stapled peptide structure prediction. The support vector machine model performed best, while two deep learning models achieved the highest accuracy of 1.0 on an external test set. Designed cysteine‐ and lysine‐stapled peptides inhibited various bacteria with low concentrations and showed good serum stability and low haemolytic activity. This study highlights the potential of the deep learning method in peptide modification and design.

## Graphical abstract

Antimicrobial peptides (AMPs) face stability and toxicity challenges in clinical use. Stapled modification enhances their stability and effectiveness, but its application in peptide design is rarely reported. This study built 10 prediction models for stapled AMPs using deep and machine learning, tested their accuracy with an independent data set and wet lab experiments, and characterised stapled loop structures using structural, sequence and amino acid descriptors. AlphaFold improved stapled peptide structure prediction. The support vector machine model performed best, while two deep learning models achieved the highest accuracy of 1.0 on an external test set. Designed cysteine‐ and lysine‐stapled peptides inhibited various bacteria with low concentrations and showed good serum stability and low haemolytic activity. This study highlights the potential of the deep learning method in peptide modification and design.

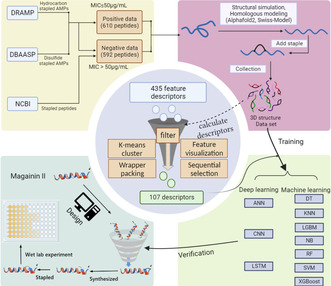

## 1 Introduction

Excessive antibiotic use has led to the global emergence of bacterial resistance, which poses a significant threat to public health (De Oliveira et al. 2020). Antimicrobial peptides (AMPs), also referred to as host‐defence peptides (HDPs), are components of innate immunity in microorganisms (Hancock and Sahl 2006; Mangoni et al. 2016). They possess broad‐spectrum antimicrobial activity and a limited propensity for resistance, thus gaining attention as potential clinical alternatives to conventional antibiotics (Browne et al. 2020; Lemaitre et al. 1996). In recent years, numerous AMPs have been identified across a wide range of multicellular organisms, and significant advancements in peptide synthesis technology have greatly facilitated the design and discovery of synthetic AMPs (Fan et al. 2016; Kang et al. 2019). The number of AMPs being developed has surged, and the latest version of the DRAMP (Data Repository of Antimicrobial Peptides) database contains approximately 30,000 natural or synthetic AMPs (Shi et al. 2022). However, relatively few AMPs have entered the clinical development stage (Browne et al. 2020). The primary challenge hindering the progress of AMPs is their susceptibility to environmental factors. For instance, AMP activity is vulnerable to proteases present in the serum, variations in pH or salt concentrations. Moreover, at high therapeutic doses, AMPs may exhibit haemolytic activity (Zhang et al. 2022).

Stapling of peptides has become a rational strategy in peptide modification studies in recent years (Stone et al. 2018; You et al. 2023), and the fundamental approach is to link the amino acid residues of short peptides using chemical bonds and tiny molecules (Kumar et al. 2018). Stapled hydrocarbons have been demonstrated to enhance the stability of peptides and optimise their bioactivity (Bird et al. 2016, 2011; Walensky and Bird 2014). Mourtada et al. (2019) designed a double‐hydrocarbon‐stapled AMP that could kill multidrug‐resistant gram‐negative pathogens in a mouse model with no observed toxicity in murine studies. They attached staples at different Magainin II positions and replaced the adjacent amino acid residues with unnatural amino acids to form a staple structure. Li et al. (2020) chose the alkylation of the ε‐amino group of the lysine residue as the key cyclization step (Olsen et al. 2005; Fukuyama et al. 1995). The designed stapled peptides improved the stability of protease hydrolysis, although some had higher antimicrobial activities than those of linear AMPs. In another study by Li et al. (2020), hydrocarbon chains were stapled separately to five different AMPs to obtain LS‐BF1 and LS‐BF3 with substantially improved plasma stability and reduced cytotoxicity (Hu et al. 2022). In our previous study, 181 stapled AMPs were collected and deposited in the third version of the DRAMP database (Fan et al. 2016; Kang et al. 2019; Shi et al. 2022), which can be freely accessed at http://dramp.cpu‐bioinfor.org/. However, the rational design of stapled AMPs remains a significant challenge. To add staples, the amino acid residues in AMPs are often mutated, which may affect the activity of AMPs. The mechanism of the antimicrobial activity of stapled peptides is unclear; large amounts of synthesised stapled AMPs are required for experimental determination. Therefore, predictive models that can accelerate the rational design and selection of stapled peptides are necessary.

Recent advances in machine learning and artificial intelligence have accelerated the design of AMPs. Machine learning can significantly increase efficiency while reducing experimental and financial expenditure (Porto et al. 2018; Wang et al. 2022). mACPpred (Boopathi et al. 2019) used seven feature encodings based on composition, physicochemical properties and profiles as input for a support vector machine (SVM) to develop a method for predicting anticancer peptides, with an accuracy of 0.914. Wang et al. (2021) constructed a long short‐term memory (LSTM)‐based deep‐learning prediction model, indicating that it is feasible to artificially divide a data set according to the strength of the antimicrobial activity and use it for machine learning. Hussain (2022) compared two convolutional DNN models and established sAMP‐PFPDeep to predict short AMPs, which included three channels comprising information related to the position, frequency and sum of 12 physicochemical features. An accuracy of 87.37%, better than those of the state‐of‐the‐art methods, including Deep‐AmPEP30 (with an accuracy of 82.56%), was obtained (Yan et al. 2020). Although AMP prediction using machine learning has made significant progress in recent years, only a few studies have predicted specifically modified peptides. Currently, most peptide prediction methods are based on the sequence and/or physicochemical properties of amino acids in peptides and cannot characterise the effects of stapled peptides. Therefore, existing prediction methods for AMPs are not suitable for predicting stapled AMPs.

The tertiary structure of a peptide is important for its biological functions. Advancements in protein structure prediction in recent years have promoted the discovery of drug targets and the exploration of protein and peptide drugs. The precision of virtual screening and molecular dynamic simulations has significantly increased because the accuracy of predicted structures is similar to those of experimentally measured ones such as RoseTTAFold (Baek et al. 2021) and AlphaFold2 (Tunyasuvunakool et al. 2021). In our previous study, we constructed a 3D structure‐based model to predict the antimicrobial activity of peptides and demonstrated its effectiveness in vitro (Liu et al. 2018). Owing to the unique spatial structure of the stapled peptides, we demonstrated that structural features can be a rational format for characterising peptide activity.

In this study, we constructed deep learning‐based models to predict the antibacterial activity of stapled AMPs. First, we created a 3D structural database of the stapled peptides and screened 107 feature descriptors related to antimicrobial activity. Based on the screened descriptors, seven traditional machine learning and three deep learning models were constructed to predict the antimicrobial activity of stapled peptides. Their performance was evaluated using a 10‐fold cross‐validation method. An external independent data set derived from a recently published study is used to test the accuracy of the models. Cysteine‐ and lysine‐stapled peptides were screened using these models; positive candidates were synthesised; and their antibacterial activities were tested in a wet laboratory experiment. Furthermore, we tested the haemolytic activity, serum stability and antimicrobial mechanism of these stapled peptides in a wet laboratory experiment. The results showed that two deep learning models, convolutional neural networks (CNNs) and LSTM, outperformed other traditional machine learning models in terms of accuracy. Moreover, lysine stapling has the potential to be a rational modification approach owing to its low haemolytic activity and high serum stability.

## 2 Materials and Methods

### 2.1 Data Collection and Splitting

The collected stapled AMPs included disulfide cyclic‐stapled peptides from DBAASP (https://dbaasp.org/home/) (Pirtskhalava et al. 2021). To conduct a detailed filtering process, utilise the ‘Search’ function with the following parameters: ‘Sequence Length’ set to ‘5–50’, ‘Complexity’ set to ‘Monomer’, ‘Unusual Amino Acid’ set to ‘Without Modification’ and the ‘Intrachain Bond’ set to ‘DSB‐Disulfide Bond’. The search was conducted based on the specified requirements.

Hydrocarbon‐stapled peptides from DRAMP (Shi et al. 2021) (http://dramp.cpu‐bioinfor.org/) and NCBI (https://www.ncbi.nlm.nih.gov/). For DRAMP, the ‘Stapled AMPs’ link on the main page of the database provides a direct avenue for searching the entirety of the stapled antimicrobial peptides amassed within the database, facilitating the retrieval of pertinent information such as the staple type, staple site and other relevant details for each stapled antimicrobial peptide. The antimicrobial activity of stapled peptides collected on NCBI was determined by reviewing the relevant literature. In the case of stapled peptides for which no clear description of antimicrobial activity could be found, their sequences were compared with those of known stapled antimicrobial peptides in DRAMP. Those with sequence inconsistencies were regarded as stapled peptides with no antimicrobial activity.

All the stapled peptides were collected between July 2022 and November 2022. The length of the stapled peptides ranged from 5 to 50 amino acids. The length of the stapled peptides ranged from 5 to 50. Stapled peptides with minimal inhibit concentration (MIC) or minimum bactericidal concentration(MBC) values for any bacteria less than or equal to 50 μg/mL were considered positive. Peptides that were not reported for definitive antimicrobial activity and had no more than 30% identity with the sequences in DRAMP and DBAASP were collected from the database as negative‐stapled peptides. Stapled peptides with MIC or MBC values greater than 50 μg/mL for any bacteria were considered negative.

**FIGURE 1:**
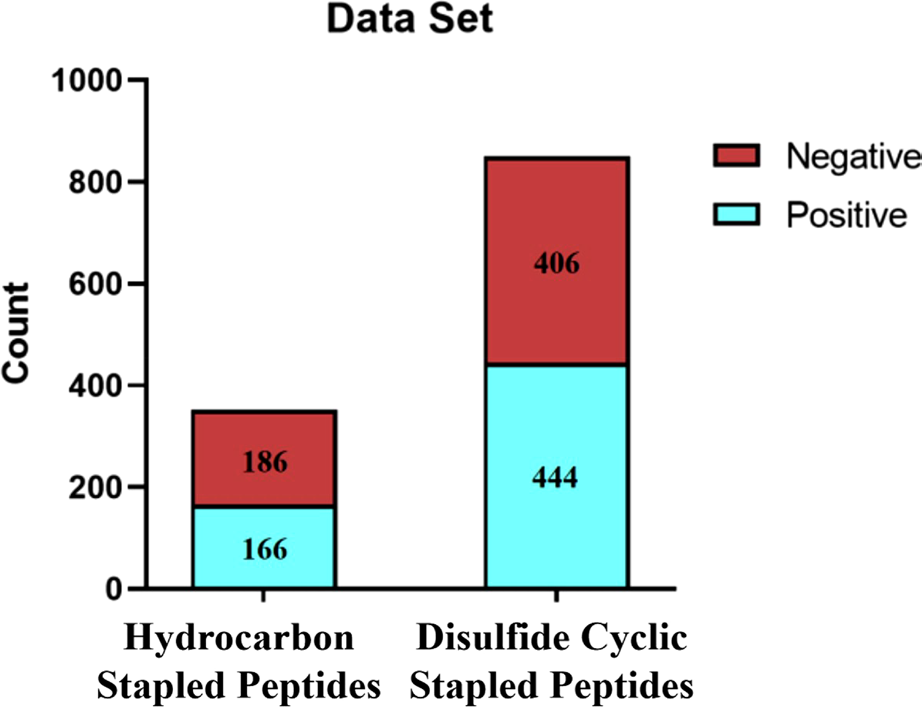
Composition of the sample data set.

As shown in Figure 1, the data set contained 1202 peptides from two groups: hydrocarbon‐stapled peptides and disulfide cyclic‐stapled peptides. The ratio of positives to negatives was approximately 1:1, with 610 and 592 samples in the positive and negative data sets, respectively.

### 2.2 Establishment of 3D Structural Data Set

Most of the stapled peptides collected did not have an experimentally determined structure. First, the peptide chain structure of the stapled peptides was simulated using Swiss‐Model (Leman et al. 2020) (stapled peptides with longer than 30 amino acids, https://swissmodel.expasy.org/) or AlphaFold2 (Tunyasuvunakool et al. 2021) (stapled peptides with less than 30 amino acids). The 3D structure of a linear peptide can be generated by inputting the amino acid sequence of a stapled peptide while using the default settings. The stapled structures were constructed by MOE software (Chemical Computing Group 2019) (Molecular Operating Environment, Chemical Computing Group, Montreal, Canada, Version: 2019) and then saved in the pdb format. The staple structures were constructed using the ‘builder’ module in MOE, and energy minimisation was conducted in default Amber10:EHT forcefield to obtain a stable conformation.

### 2.3 Extraction and Screening of Feature Descriptors

A total of 435 descriptors were calculated for the stapled peptides in the 3D structural data set using MOE (Chemical Computing Group 2019). The obtained feature descriptor data were standardised in the use of ‘StandardScaler’ in the scikit‐learn package, and the missing values were denoted by ‘0’. Wrapper packing, sequential feature selection, k‐means cluster analysis and feature visualisation analysis are used to reduce the dimensionality of the feature data. Wrapper packing and sequential features were processed through the Scikit‐learn package in Python (version 3.11). The K‐means clustering analysis was performed using the elbow method to obtain optimal clusters of features. Feature visualisation analysis was conducted using the TreeExplainer function in the SHAP package to compute the SHAP values of the features and visualise their significance with the XGBRegressor model.

### 2.4 Machine Learning Methods

Traditional machine learning algorithms, including decision tree (DT), k‐nearest neighbour (KNN), light gradient boosting machine (LGBM), naive Bayes (NB), random forest (RF), support vector machine (SVM) and extreme gradient boosting (XGB), were employed using the scikit‐learn module in Python. Additionally, three deep learning artificial neural network (ANN) models, convolutional neural networks (CNN) and long short‐term memory (LSTM) (Labute 2000; Deng et al. 2018; Zhang 2016; Song et al. 2022; Fu et al. 2020; Veltri et al. 2018) were compared and implemented using scikit‐learn, TensorFlow and Keras.

### 2.5 Evaluation and Validation of Model Performances

Each model's parameters were fine‐tuned and evaluated with a test set to achieve optimal performance. Prediction models were evaluated using the ROC (receiver operating characteristic) area under the curve (ROC‐AUC), Matthews correlation coefficient (MCC), sensitivity (Sn), specificity (Sp), precision (Pr) and accuracy (ACC) (Kha et al. 2022; Le et al. 2022). These metrics were calculated as follows:

$$\mathrm{ACC}=\frac{\mathrm{TP}+\mathrm{TN}}{\mathrm{TP}+\mathrm{TN}+\mathrm{FP}+\mathrm{FN}}$$

$$\mathrm{Pr}=\frac{\mathrm{TP}}{\mathrm{TP}+\mathrm{FP}}$$

$$\mathrm{Sn}=\frac{\mathrm{TP}}{\mathrm{TP}+\mathrm{FN}}$$

$$\mathrm{Sp}=\frac{\mathrm{TN}}{\mathrm{FP}+\mathrm{TN}}$$

$$\mathrm{MCC}=\frac{\left(\mathrm{TP}\times \mathrm{TN}\right)-\left(\mathrm{FP}\times \mathrm{FN}\right)}{\sqrt{\left(\mathrm{TP}+\mathrm{FP}\right)\times \left(\mathrm{TP}+\mathrm{FN}\right)\times \left(\mathrm{TN}+\mathrm{FP}\right)\times \left(\mathrm{TN}+\mathrm{FN}\right)}}$$

- TP, TN, FP and FN denote the numbers of true positives, true negatives, false positives and false negatives, respectively.

### 2.6 Peptide Synthesis

The peptide chains tested in this study were synthesised using solid‐phase peptide synthesis (SPPS). Disulfide cyclic‐stapled peptides were oxidised using dimethyl sulfoxide (DMSO) to link the two cysteines in the peptide chain. For cysteine hydrocarbon‐stapled peptides, 1,7‐octadecyl or 1,8‐nonadienyl was made to react with two cysteines in a peptide chain, using NMP as the synthesis medium, and the final products were produced under 365 nm UV irradiation. For lysine‐stapled peptides, a serum stability assay was conducted as described by Li, with minor modifications (Li et al. 2020). magainin II was synthesised at GL Biochem (Shanghai, China) Ltd. Disulfide cyclic‐stapled peptides were synthesised at QYAOBIO (China Peptides Co. Ltd.). Cysteine hydrocarbon‐stapled peptides were synthesised at Scier Bio Co. Ltd. Lysine‐stapled peptides were synthesised at GL BioChem Ltd. (Shanghai, China). Linear lysine peptides were synthesised at Synpeptide Co. Ltd.

### 2.7 Bacterial Strains

*Escherichia coli* ( *E. coli* , ATCC25922), *Klebsiella pneumoniae* ( *K. pneumoniae* , ATCC10031), *Staphylococcus aureus* ( *S. aureus* , ATCC6538P), *Pseudomonas aeruginosa* ( *P. aeruginosa* , ATCC27853) and *methicillin‐resistant Staphylococcus aureus* (*MRSA*) were used to evaluate the antimicrobial activity of the peptides and were purchased from BeNa Culture Collection.

### 2.8 Antimicrobial Activity Assay

The MICs of the peptides in vitro were determined using the broth microdilution method with minor modifications (Wiegand et al. 2008). Peptides were incubated with bacteria (about 5 × 10^5^ CFU/mL), and PBS was used as the negative control. After 18–24 h of incubation at 37°C, the bacteria were checked for growth by recording the values at OD_600_, with three replicates in each group.

### 2.9 Haemolytic Activity Assay

Sheep red blood cells were washed three times with PBS and resuspended in PBS. They were then incubated with specific concentrations of AMPs for 1 h at 37°C. The absorbance at 570 nm was determined by centrifuging 100 μL of the supernatant at 10^6^ × *g* at 4°C for 10 min. The negative control (0% haemolysis) was incubated in PBS with serum, and the positive control (100% haemolysis) was 0.1% Triton X‐100 incubated in serum. Haemolysis was calculated using the following formula:

$$\text{Haemolysis activity}\left(\%\right)=\frac{{\mathrm{OD}}_{\text{SAMPLE}}-{\mathrm{OD}}_{\text{NEGATIVE}}}{{\mathrm{OD}}_{\text{POSITIIVE}}-{\mathrm{OD}}_{\text{NEGATIVE}}}\times 100\%,$$

- where OD_SAMPLE_ represents the absorbance of the peptide sample at 570 nm, and OD_NERATIVE_ and OD_POSITIVE_ represent the absorbance at 0% and 100% haemolysis, respectively, as determined in PBS and 0.1% Triton X‐100. Each test was repeated at least three times using three replicates.

### 2.10 Serum Stability Assay

The serum stability assay was conducted as described by Li et al. (2020) with minor modifications. In brief, each peptide was dissolved in PBS solution containing 25% foetal bovine serum (FBS) at a final concentration of 2048 μg/mL and diluted after incubation for 8 h. *E. coli* was used as the bacterium for the stability assay, and the bacterial inhibition experiment was performed according to the bacterial inhibitory activity assay. Residual activity was calculated using the following formula:

$$\text{Residual activity}={\mathrm{MIC}}_{\text{before}}/{\mathrm{MIC}}_{\text{after}}$$

- where MIC_before_ represents the MICs against *E. coli* before incubation, and MIC_after_ represents the MICs against *E. coli* after incubation for 8 h.

### 2.11 Circular Dichroism (CD) Spectra Assay

Peptides were extracted and dissolved in phosphate buffered saline (PBS) with 100 mM sodium dodecyl sulfate (SDS) to a concentration of 0.1 mg/mL. The dissolved peptide solution and the blank control solution were utilised to ascertain the circular dichroism (CD) spectra between 190 nm and 260 nm with a data interval of 0.4 nm using a Jasco J‐810 spectrophotometer in a 1‐mm quartz dish at 25°C.

### 2.12 Scanning Electron Microscopy (SEM) Assay

*S. aureus* were cultured to reach the logarithmic phase in an MH broth medium. Following three washes with PBS by centrifugation at 3800 rpm for 5 min, the samples were diluted to approximately 1 × 10^8^ CFU/mL and treated with specific concentrations of peptides for 2 h at 37°C. Subsequently, the supernatant was discarded, and the precipitate was washed three times with PBS. The precipitate was then dehydrated in different concentrations of ethanol (50%, 70%, 90% and 100%) sequentially for 10 min at 4°C after fixation in 2. The samples were then treated with 5% (w/v) glutaraldehyde for 10 min, followed by immersion in 100% ethanol for 15 min. Subsequently, a mixture of 100% ethanol and tertiary butyl alcohol (1:1) was used for an additional 15 min, and then the samples were incubated in anhydrous tert‐butanol for 10 min. Following drying, the samples were coated with gold palladium, and changes in bacterial morphology were observed using a scanning electron microscope.

## 3 Results

**FIGURE 2:**
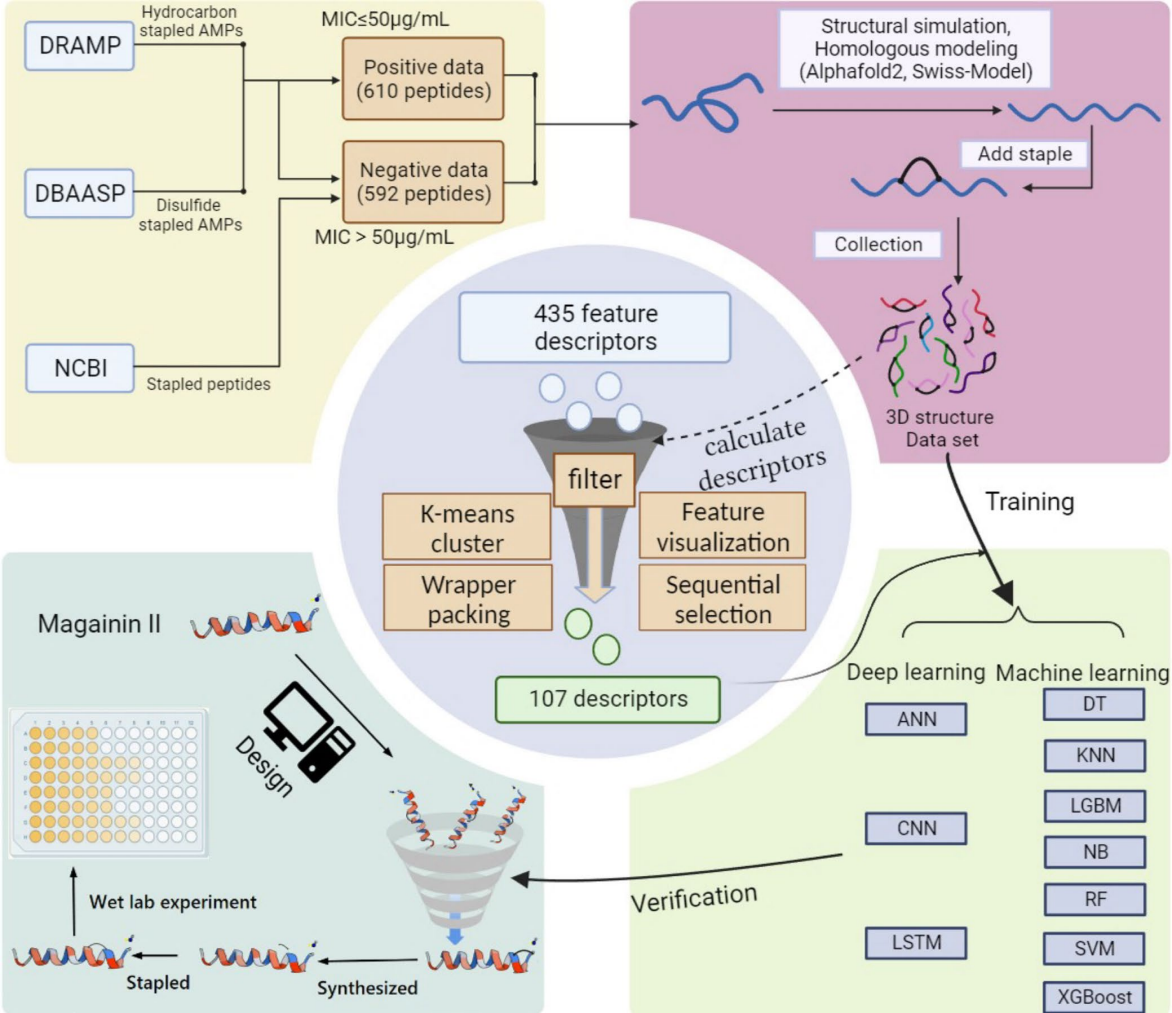
The overall construction and validation flowchart.

The construction and validation flowchart is shown in Figure 2.

### 3.1 Descriptor Filtering

After self‐iterative processing, the wrapper packing method demonstrated an optimal performance with a feature count of 70, achieving a model accuracy of 0.813. The sequential selection approach used forward iterations and achieved the highest accuracy of 0.805 with 150 features. Table 1 presents a comparison of the model output data from the two methods.

**TABLE 1:** Model output of wrapper package method and sequential selection method.

	Features	ACC	MCC	Pr	Sn	Sp	AUC
Wrapper packing	70	0.813	0.626	0.809	0.802	0.824	0.890
Sequential selection	150	0.805	0.611	0.785	0.819	0.792	0.881

After feature comparison, the intersection of the screening results from the two methods was determined, resulting in the selection of 32 features. Supporting Information S1 lists the intersections of the output features from both data sets and their corresponding meanings.

**FIGURE 3:**
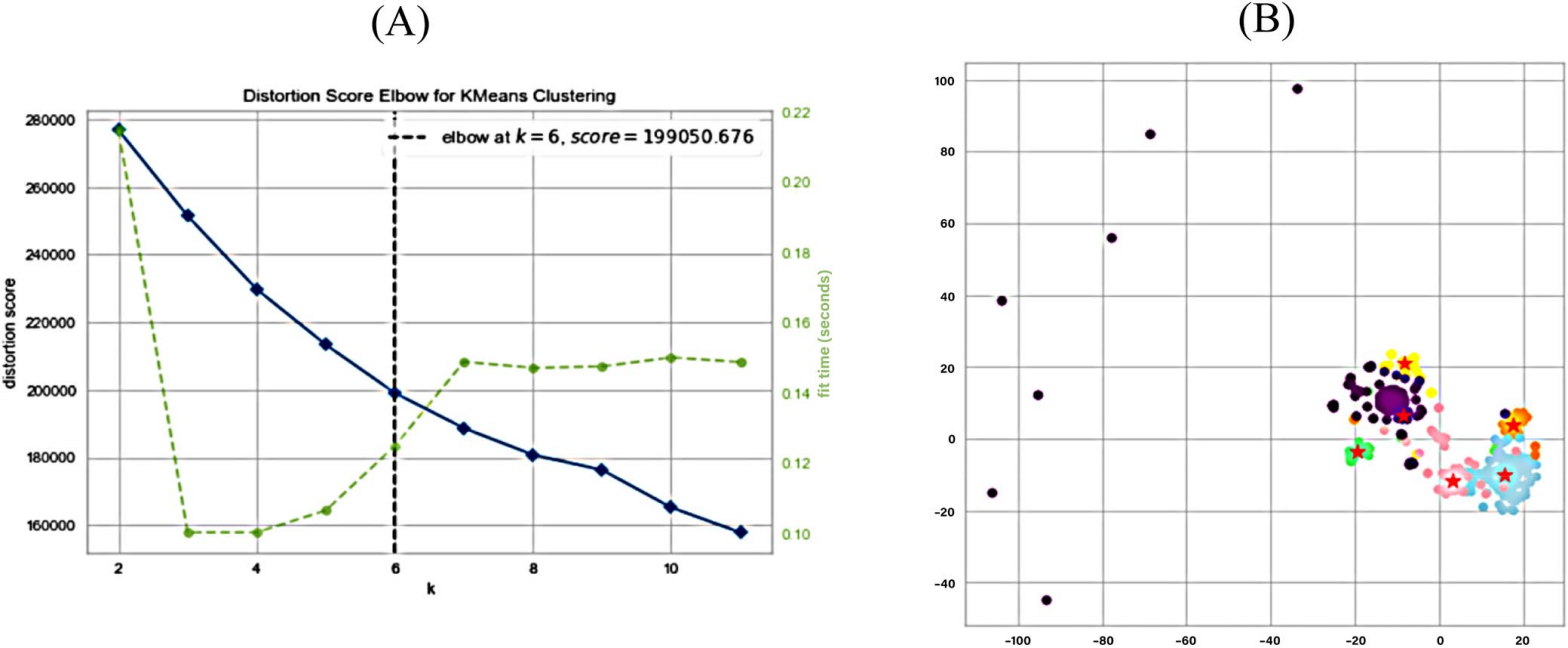
(A) K‐means elbow chart for 435 feature descriptors; (B) Visualisation of K‐means clustering analysis for feature descriptors. The pentagram symbol is represented as the cluster centre.

**FIGURE 4:**
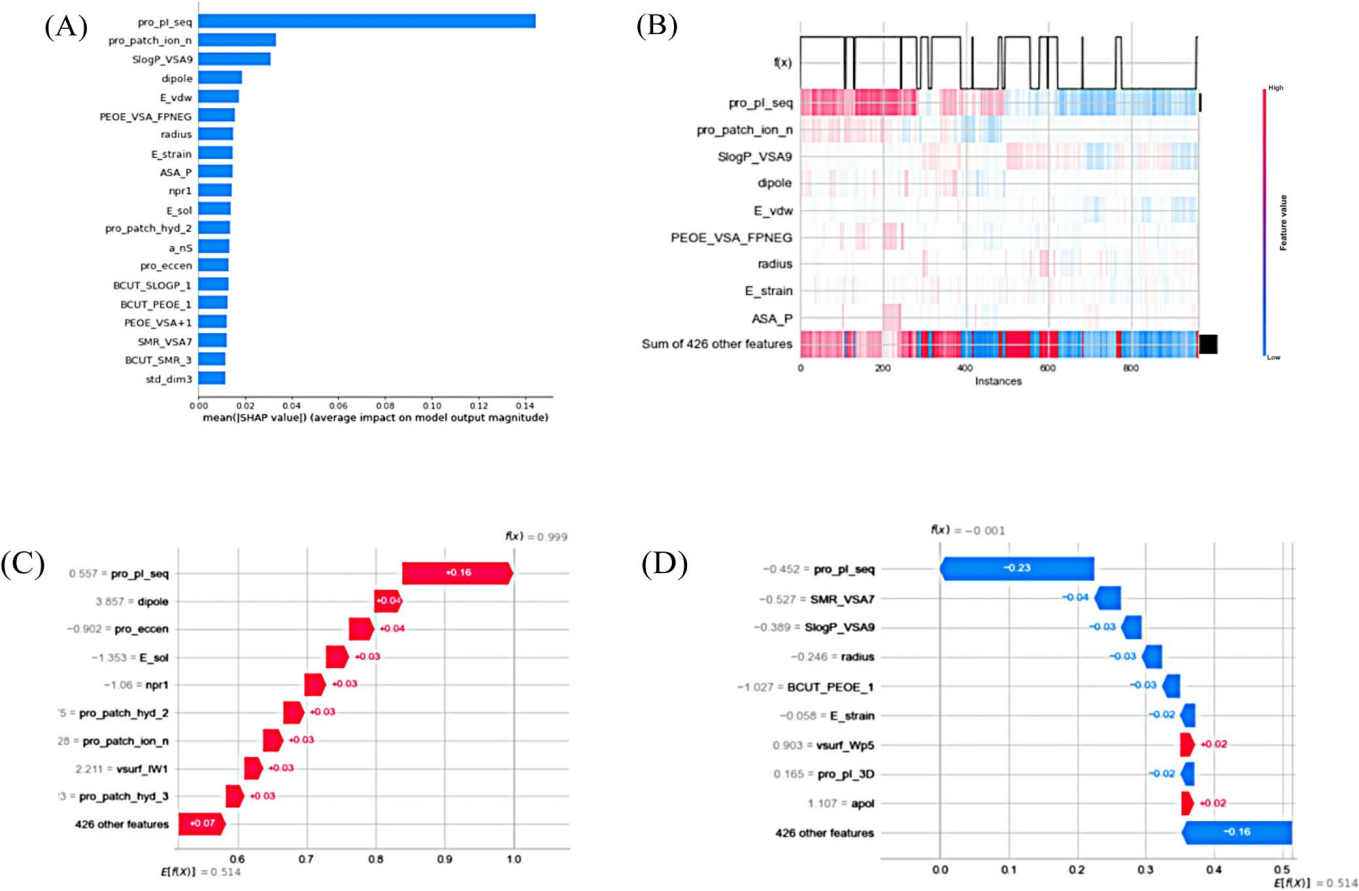
(A) Sorting results of feature importance. Bar plot with mean SHAP absolute value as the X axis and descriptor feature as the Y axis. (B) SHAP value heat map of characteristics. The X axis represents instances, the Y axis represents features, and the right colour scale encodes the SHAP value. Above the heatmap matrix is the model output, the grey line is the baseline, and on the right side is the global importance of each feature input. (C) The characteristic contribution values of peptides predicted by the model to be positive and their ranking results. (D) The characteristic contribution values of peptides predicted by the model to be negative and their ranking results.

The 435 feature descriptors were classified into six categories using K‐means clustering (Figure 3A,B, the classification results are shown in Supporting Information S2 and S3). Visualising the relative importance of each feature by Shapley additive explanations (SHAP, Figure 4A), the protein sequence isoelectric point (pro_pI_seq) had the greatest impact on the model's antimicrobial activity prediction results and was more important than the other features. The feature descriptors protein ion patch area (pro_patch_ion_n) and lipid‐water partition coefficient (SlogP_VSA9), ranked second and third, respectively, and also had higher mean absolute values of SHAP than those of the remaining features, indicating relatively high importance (Figure 4B). SHAP also shows the potential correlation between the antimicrobial activity of the samples and feature descriptors, such as radius, BCUT_SLOGP_1 (BCUT_SLOGP_1 descriptors using atomic contribution to logP), BCUT_PEOE_1 (BCUT_PEOE_1 descriptors are calculated from the eigenvalues of a modified adjacency matrix) and BCUT_SMR_3 (BCUT_SMR_3 descriptors using atomic contribution to molar refractivity) (Figure 4C).

Various characteristics have varying significance in predicting specific peptides. Some features played an important role in reducing the predicted value (Figure 4D). The peptide with the highest SHAP value among these features can be considered an antimicrobial peptide. However, single descriptor features have a very limited impact on the model output results. Model prediction of the antimicrobial activity of the samples is a result of the combined effect of the factors. Thus, the prediction of sample antibacterial activity is a collective impact of various factors (Supporting Information S4).

By combining the wrapper packing method, sequential feature method, K‐means clustering algorithm, and SHAP interpretable machine learning for the screening of the feature data set, significant characteristics were evaluated, causing a reduction in the size of the initial feature data set. We also incorporated our experience in screening the features of antimicrobial peptides based on hydrophobicity, side‐chain volume, polarity, pH at the isoelectric point, dissociation constants, net charge and other attributes. Finally, 107 features (Supporting Information S5) were screened to construct the prediction model.

### 3.2 Estimating the Performance of Training Models

**FIGURE 5:**
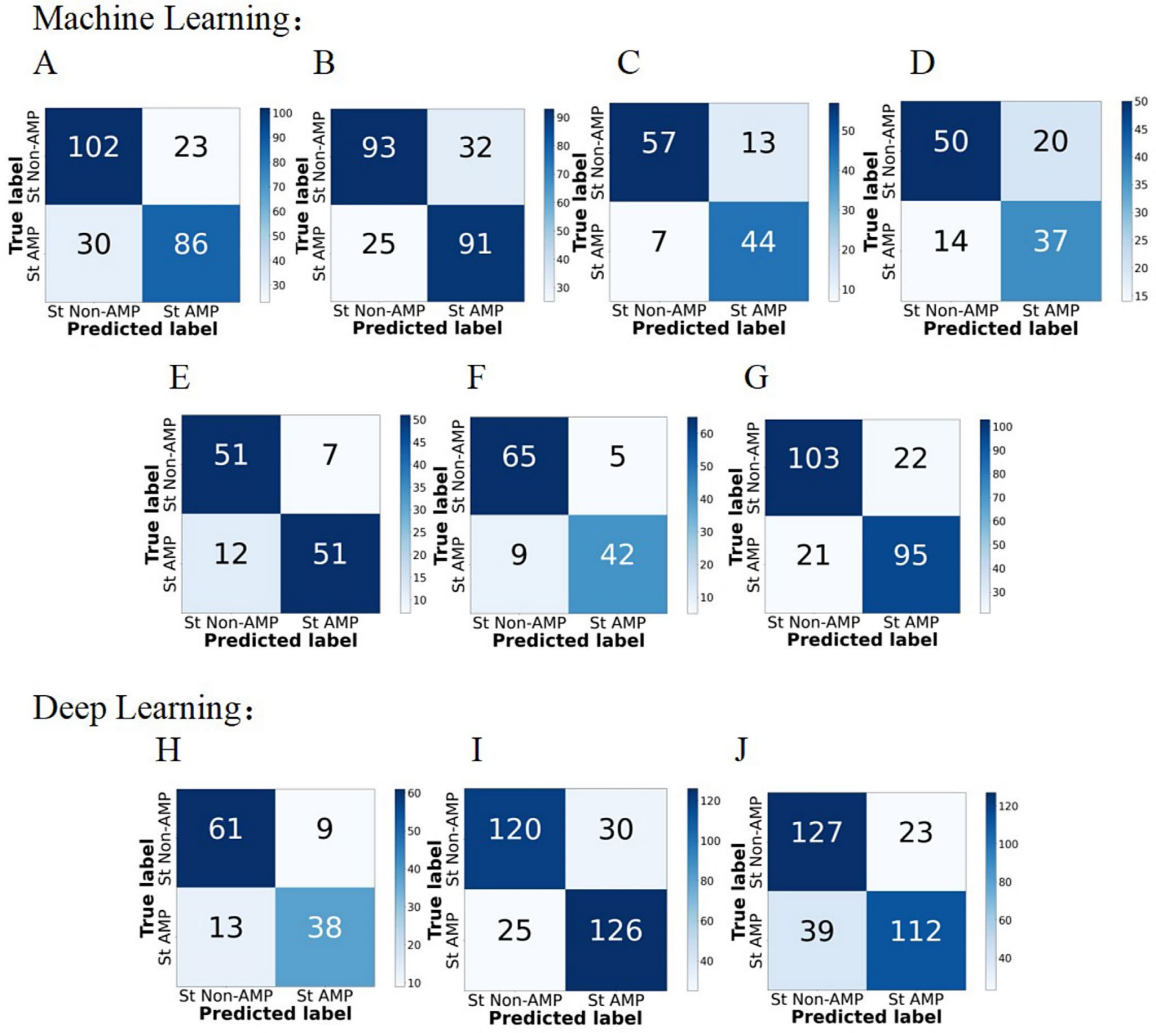
Confusion matrices for 10‐fold cross‐validation set testing of machine learning: (A) DT, (B) KNN, (C) LGBM, (D) NB, (E) RF, (F) SVM, (G) XGB and deep learning: (H) ANN, (I) CNN and (J) LSTM.

**FIGURE 6:**
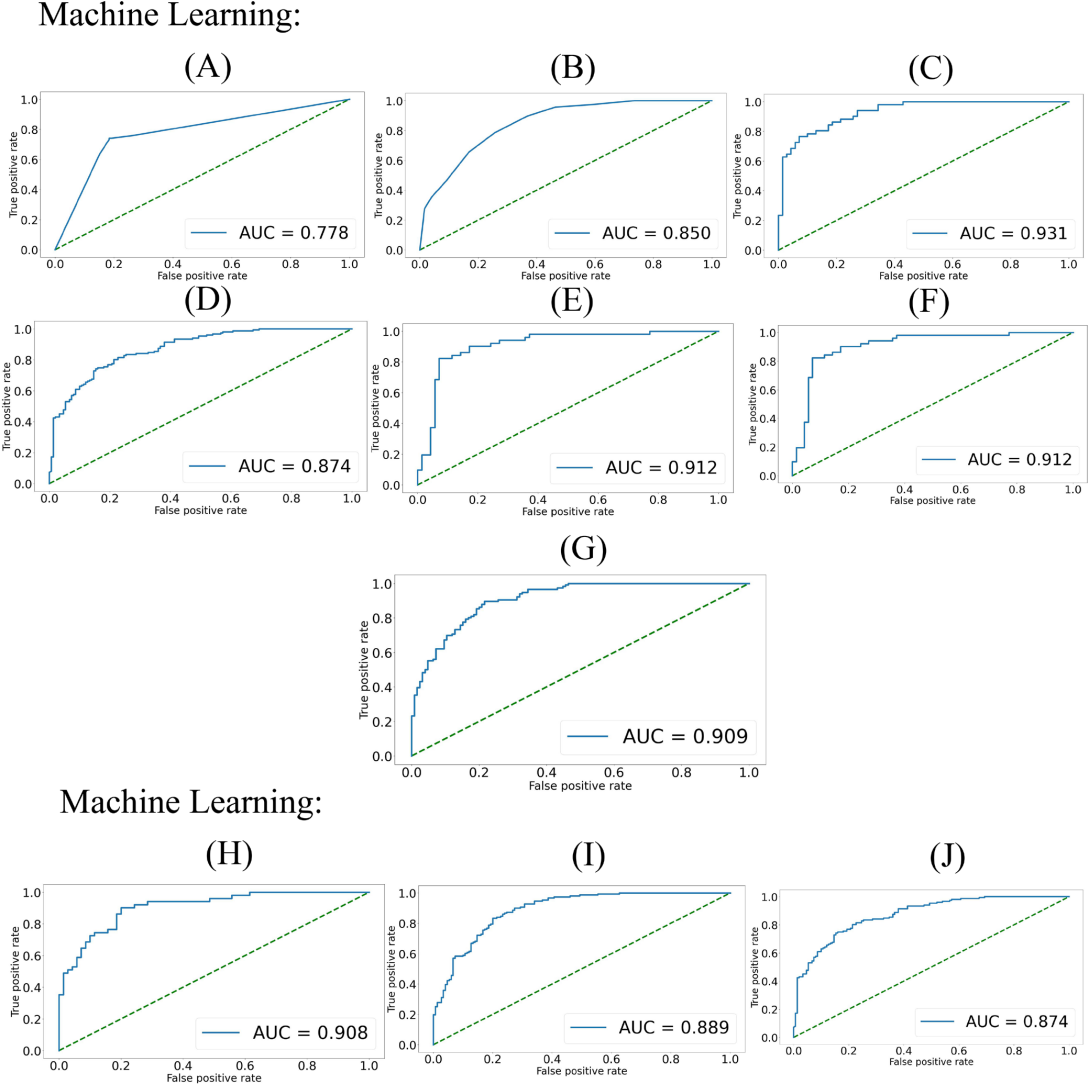
ROC‐AUC for 10‐fold cross‐validation set testing of machine learning: (A) DT, (B) KNN, (C) LGBM, (D) NB, (E) RF, (F) SVM, (G) XGB and deep learning: (H) ANN, (I) CNN and (J) LSTM.

Based on the screened 107 features, we trained and evaluated the performance of seven traditional machine learning models and three deep learning models. The evaluation results were based on the optimal values obtained by adjusting the parameters of each model (the process of parameter adjustment and selection for each model is described in Supporting Information S6), as listed in Table 2. The confusion matrices (Figure 5) and ROC‐AUC (Figure 6) show the 10 machine learning models performance in the use of 10‐fold cross‐validation. Among the six assessment indicators, SVM was ranked first for four indicators and fourth for one. The LGBM exhibited the highest Sn and AUC. Regarding deep learning, there was not much difference in the performance among the three models. LSTM (obtained first in three indicators) outperformed the other two deep‐learning models but was not as good as SVM.

**TABLE 2:** Comparative analysis of different machine‐learning models using the 10‐fold cross‐validation method.

Models	Traditional machine learning							Deep learning		
	DT	KNN	LGBM	NB	RF	SVM	XGB	ANN	CNN	LSTM
ACC	0.780	0.763	0.835	0.719	0.843	**0.884**	0.822	0.818	0.817	0.794
MCC	0.560	0.528	0.670	0.435	0.689	**0.762**	0.644	0.625	0.634	0.592
Pr	0.789	0.740	0.772	0.649	0.879	**0.894**	0.802	0.809	0.807	0.830
Sn	0.741	0.784	**0.863**	0.726	0.810	0.824	0.836	0.745	0.834	0.742
Sp	0.816	0.744	0.814	0.714	0.879	**0.929**.	0.808	0.871	0.800	0.847
AUC	0.778	0.850	**0.931**	0.803	0.912	0.912	0.909	0.908	0.889	0.874

*Note:* The optimal model for each evaluation metric is given in boldface.

### 3.3 Model Validation by External Independent Data Set

To test the performance of the models, we used recently published data from Hu et al. (2022) as an external independent data set. This data set included 13 stapled peptides that were not part of our training data and showed strong antimicrobial activity against both gram‐positive and gram‐negative bacteria. Ten models were used to predict the antimicrobial activity of the stapled peptides, and the score predicted by each model ranged from 0 to 1, with a score greater than 0.5 considered to indicate antimicrobial activity.

The prediction results of the 10 models for the 13 experimentally verified stapled AMPs are listed in Table 3. On the independent test set, two of the three deep learning models, CNN and LSTM, outperformed all the traditional machine learning models. Another deep learning model, the ANN, produced a false‐negative result for the LS‐PTa peptide. Interestingly, none of the traditional machine learning models, including SVM, predicted LS‐PTa activity correctly. This indicates that deep learning methods may uncover features that traditional machine learning methods cannot detect, leading to more precise predictions.

**TABLE 3:** Prediction results of external independent data set using 10 models.

ID	Stapled type	Sequences	Result^a^	Traditional machine learning							Deep learning		
				DT	KNN	LGBM	NB	RF	SVM	XGB	ANN	CNN	LSTM
LS‐PTa	(E)‐but‐2‐enyl	FFGSVLⓀLIPⓀIL‐NH_2_	Positive	(−^b^)/F	(−)/F	(−)/F	(−)/F	(−)/F	(−)/F	(−)/F	(−)/F	(+^c^)/T	(+)/T
LS‐AP1		GFKDLLKGAAⓀALVⓀTVLF‐NH_2_	Positive	(−)/F	(+)/T	(−)/F	(−)/F	(−)/F	(+)/T	(−)/F	(+)/T	(+)/T	(+)/T
LS‐AP2		GFKDLLⓀGAAⓀALVKTVLF‐NH_2_	Positive	(−)/F	(+)/T	(−)/F	(−)/F	(−)/F	(+)/T	(−)/F	(+)/T	(+)/T	(+)/T
LS‐AP3		GFⓀDLLⓀGAAKALVKTVLF‐NH_2_	Positive	(−)/F	(+)/T	(−)/F	(−)/F	(−)/F	(+)/T	(−)/F	(+)/T	(+)/T	(+)/T
LS‐DAS		FFGKVLⓀLIRⓀIF‐NH_2_	Positive	(+)/T	(−)/F	(+)/T	(−)/F	(+)/T	(+)/T	(+)/T	(+)/T	(+)/T	(+)/T
LS‐PaD1		PKINLⓀILGⓀILRLAAAFK‐NH_2_	Positive	(+)/T	(+)/T	(+)/T	(+)/T	(+)/T	(+)/T	(+)/T	(+)/T	(+)/T	(+)/T
LS‐PaD2		PⓀINLⓀILGKILRLAAAFK‐NH_2_	Positive	(+)/T	(+)/T	(+)/T	(+)/T	(+)/T	(+)/T	(+)/T	(+)/T	(+)/T	(+)/T
LS‐BF1		VKRFKⓀFFRⓀFKKFV‐NH_2_	Positive	(−)/F	(+)/T	(+)/T	(+)/T	(+)/T	(+)/T	(+)/T	(+)/T	(+)/T	(+)/T
LS‐BF2		VⓀRFKⓀFFRKFKKFV‐NH_2_	Positive	(−)/F	(+)/T	(+)/T	(+)/T	(+)/T	(+)/T	(+)/T	(+)/T	(+)/T	(+)/T
LS‐BF3	1,2‐bismethylenebenzene	VKRFKⓀFFRⓀFKKFV‐NH_2_	Positive	(−)/F	(+)/T	(+)/T	(+)/T	(+)/T	(+)/T	(+)/T	(+)/T	(+)/T	(+)/T
LS‐BF4		VⓀRFKⓀFFRKFKKFV‐NH_2_	Positive	(−)/F	(+)/T	(+)/T	(+)/T	(+)/T	(+)/T	(+)/T	(+)/T	(+)/T	(+)/T
LS‐BF5	but‐2‐ynyl	VKRFKⓀFFRⓀFKKFV‐NH_2_	Positive	(−)/F	(+)/T	(+)/T	(+)/T	(+)/T	(+)/T	(+)/T	(+)/T	(+)/T	(+)/T
LS‐BF6		VⓀRFKⓀFFRKFKKFV‐NH_2_	Positive	(−)/F	(+)/T	(+)/T	(+)/T	(+)/T	(+)/T	(+)/T	(+)/T	(+)/T	(+)/T
Accuracy				0.231	0.846	0.692	0.615	0.692	**0.923**	0.692	**0.923**	**1.000**	**1.000**

*Note:* Models with an accuracy rate exceeding 0.9 when validated using an external independent validation set are given in boldface.

Abbreviations: F. False; T, True.

^a^ Stapled AMP with high antimicrobial activity in the experiment results in Hu et al. (2022).

^b^ Predict a score lower than 0.5.

^c^ Predict a score of higher than 0.5.

### 3.4 Laboratory Confirmation

In this study, we designed cysteine‐stapled peptides based on Mag2 and predicted their antibacterial activities. First, methionine in the Mag2 sequence (GIGKFLHSAKKFGKAFVGEIMNS) was mutated to leucine to obtain the sequence GIGKFLHSAKKFGKAFVGEILNS. Next, two amino acid residues separated by three (i + 4) or six (i + 7) residues were modified to cysteines and linked together with disulfide bonds or hydrocarbons (octane for the i + 4 and decane for the i + 7 residue). This way, 70 stapled candidate peptides were designed. Supporting Information S6 and S7 show the sequences of the 35 disulfide cyclic‐stapled peptides and 35 hydrocarbon‐stapled peptides, respectively. The stapled peptides designed in this study were named with reference to existing research (Mourtada et al. 2019). The suffixes ‘Dc’ and ‘St’ denote disulfide cyclic‐stapled and hydrocarbon cysteine‐stapled peptides, respectively.

The designed stapled peptides were screened using the 10 models. The prediction scores for the designed 35 disulfide cyclic‐cysteine‐stapled peptides and 35 hydrocarbon–cysteine‐stapled peptides are shown in Supporting Information S7 and S8, respectively. Additionally, we developed a set of lysine‐stapled peptides that resembled an external independent data set. The sequences of the lysine‐stapled peptides and model prediction scores are presented in Supporting Information S9. Ⓚ indicates that the two amino acids were covalently bound to (E)‐but‐2‐enyl.

From the designed 70 stapled peptides, 10 of them were selected based on the screening results of prediction models and used for further laboratory validation. Table 4 displays the prediction results by different models.

**TABLE 4:** Predict the result of screened disulfide cyclic‐stapled peptides using 10 models.

ID	Traditional machine learning							Deep learning		
	DT	KNN	LGBM	NB	RF	SVM	XGB	ANN	CNN	LSTM
Mag(i + 4)14Dc	(+)^a^	(+)	(−)^b^	(+)	(+)	(+)	(−)	(+)	(+)	(+)
Mag(i + 4)15Dc	(+)	(+)	(−)	(+)	(+)	(+)	(−)	(+)	(+)	(−)
Mag(i + 4)17Dc	(+)	(+)	(+)	(+)	(+)	(+)	(−)	(+)	(+)	(−)
Mag(i + 4)0St	(+)	(+)	(+)	(+)	(+)	(+)	(−)	(+)	(+)	(+)
Mag(i + 7)11St	(−)	(+)	(+)	(+)	(+)	(+)	(+)	(+)	(+)	(+)
GAN‐P2‐2‐St	(−)	(+)	(+)	(−)	(+)	(−)	(+)	(−)	(+)	(+)
GAN‐P2‐3‐St	(−)	(+)	(+)	(−)	(+)	(−)	(+)	(−)	(+)	(+)
GAN‐P2‐4‐St	(−)	(+)	(+)	(−)	(+)	(+)	(+)	(−)	(+)	(+)
VAE‐P3‐3‐St	(+)	(+)	(+)	(+)	(+)	(+)	(+)	(+)	(+)	(+)
VAE‐P3‐5‐St	(+)	(+)	(+)	(+)	(+)	(+)	(+)	(+)	(+)	(+)

^a^ Predict a score higher than 0.5.

^b^ Predict a score of lower than 0.5.

**FIGURE 7:**
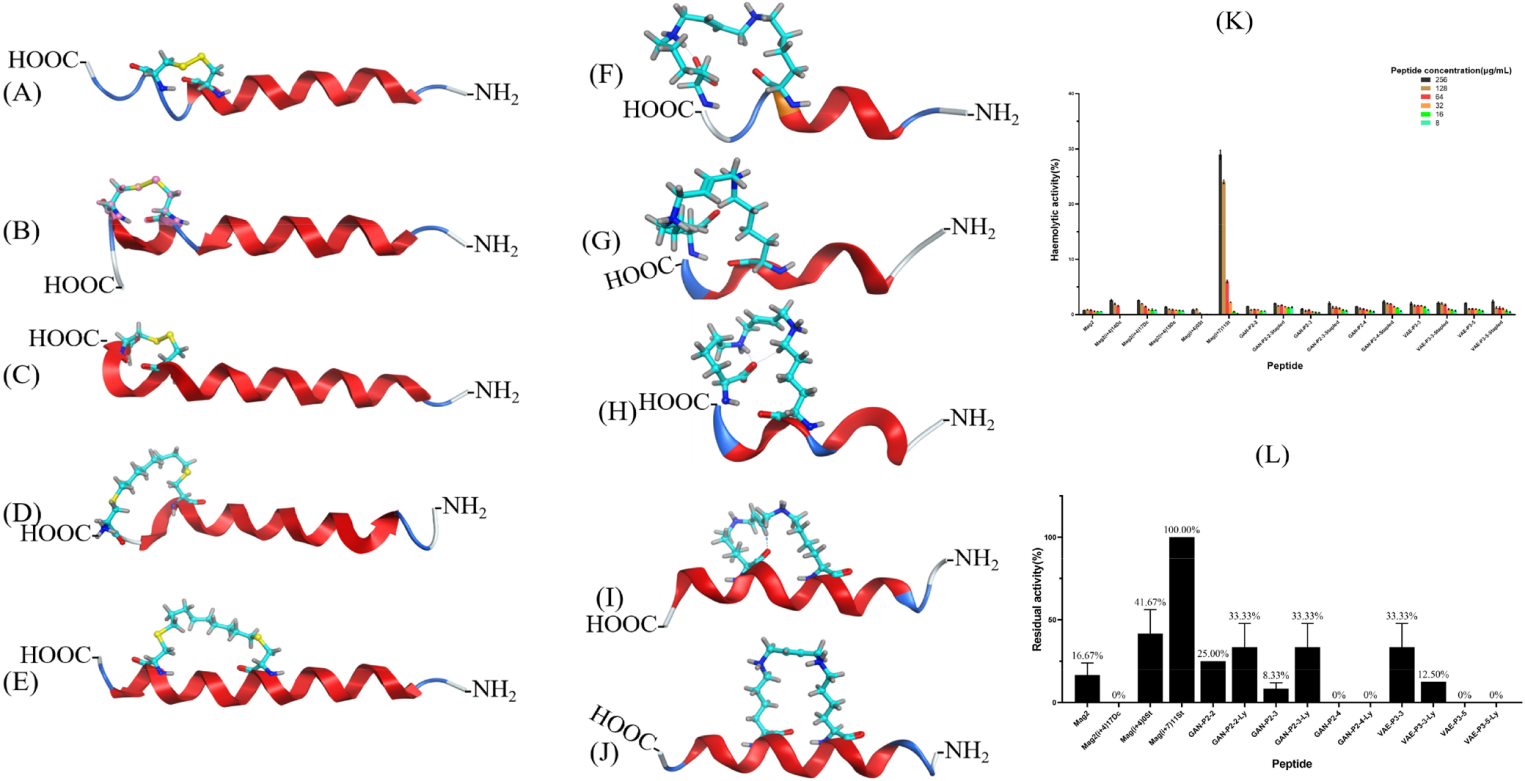
Structure of (A) Mag2(i + 4)14Dc, (B) Mag2(i + 4)15Dc, (C) Mag2(i + 4)17Dc, (D) Mag(i + 4)0St, (E) Mag(i + 7)11St, (F) GAN‐P2‐2‐St, (G) GAN‐P2‐3‐St, (H) GAN‐P2‐4‐St, (I) VAE‐P3‐3‐St and (J) VAE‐P3‐5‐St. (K) Haemolytic activity of 10 designed stapled peptides and their linear peptides. (L) Residual activity of designed stapled peptides and their linear peptides.

Based on the predicted results, 10 stapled peptides, Mag2(i + 4)14Dc, Mag2(i + 4)15Dc, Mag2(i + 4)17Dc, Mag(i + 4)0St, Mag(i + 7)11St, GAN‐P2‐2‐St, GAN‐P2‐3‐St, GAN‐P2‐4‐St, VAE‐P3‐3‐St and VAE‐P3‐5‐St, and their linear peptides were synthesised and evaluated for their antimicrobial activities against different bacterial species. The structures of the 10 designed stapled peptides are shown in Figure 7. The antimicrobial activities of stapled peptides varied compared to linear peptides (Table 5, purity and structure of synthetic peptides are shown in Supporting Information S10 and S11).

**TABLE 5:** Antimicrobial activity of AMPs in the wet lab experiments.

ID	MIC^a^ (μg/mL)				
	Gram‐negative bacteria			Gram‐positive bacteria	
	*E. coli*	*K. pneumoniae*	*P. aeruginosa*	*S. aureus*	*MRSA*
Mag2	32	16	128	16	> 128
Mag2(i + 4)14Dc	64	32	64	16	N/A
Mag2(i + 4)15Dc	512	256	> 512	> 512	N/A
Mag2(i + 4)17Dc	16	16	64	8	N/A
Mag(i + 4)0St	4	16	32	32	N/A
Mag(i + 7)11St	16	8	32	2	N/A
GAN‐P2‐2	8	4	16	8	128
GAN‐P2‐2‐St	16	1	32	2	8
GAN‐P2‐3	16	4	32	8	128
GAN‐P2‐3‐St	16	1	32	2	8
GAN‐P2‐4	8	4	32	8	64
GAN‐P2‐4‐St	16	2	16	4	16
VAE‐P3‐3	16	2	16	4	16
VAE‐P3‐3‐St	16	1	16	4	4
VAE‐P3‐5	16	1	4	2	8
VAE‐P3‐5‐St	16	4	32	4	16

^a^ MIC indicated to inhibit 99.9% of the bacterial growth.

Among the disulfide cyclic‐cysteine‐stapled peptides, Mag2(i + 4)17Dc had slightly higher antimicrobial activity than that of Mag2(i + 4)14Dc. However, Mag2(i + 4)15Dc did not exhibit any antimicrobial activity against any of the four bacterial species tested. As to hydrocarbon–cysteine‐stapled peptides, both had stronger antimicrobial activities than that of Mag2, with the MIC value of Mag(i + 4)0St being 4 μg/mL against *E. coli* and those of Mag(i + 7)11St MIC being 8 μg/mL and 2 μg/mL for *K. pneumoniae* and *S. aureus* , respectively. The MICs of both hydrocarbon–cysteine‐stapled peptides against *P. aeruginosa* were 32 μg/mL. Overall, the two hydrocarbon–cysteine‐stapled peptides showed significantly higher antimicrobial activity than those of the disulfide cyclic‐stapled peptides.

For lysine‐stapled peptides, all five stapled antimicrobial peptides exhibited high antimicrobial activity. Only the activity of VAE‐P3‐5‐St was somewhat lower than that of its linear counterpart. The antimicrobial activity of GAN‐P2‐2‐St, GAN‐P2‐3‐St, GAN‐P2‐4‐St and VAE‐P3‐3‐St against *K. pneumoniae* increased by 2–4‐fold. The MICs of GAN‐P2‐2‐St, GAN‐P2‐3‐St and VAE‐P3‐3‐St against *K. pneumoniae* reached 1 μg/mL and GAN‐P2‐4‐St reached 2 μg/mL. For *S. aureus* , the antimicrobial activities of GAN‐P2‐2‐St and GAN‐P2‐3‐St increased 4‐fold. However, the designed lysine‐stapled peptides did not significantly affect the antimicrobial activity against *E. coli* and *P. aeruginosa* .

Furthermore, we tested the antimicrobial activity of the lysine‐stapled peptides against *MRSA*. The results indicated that the antibacterial activity of GAN‐P2‐2‐St and GAN‐P2‐3‐St increased by 16‐fold to 8 μg/mL, whereas the antibacterial activity of GAN‐P2‐4‐St and VAE‐P3‐3‐St increased by 4‐fold, with that of VAE‐P3‐3‐St reaching 4 μg/mL. Our study showed that stapled antimicrobial peptides exhibited higher antibacterial activity compared to the linear peptides. This suggests that stapled antimicrobial peptides may be more effective against drug‐resistant bacteria. In addition, the stapled structure may reduce the impact of bacterial drug resistance. However, we compared only stapled and linear peptide activities and did not evaluate other factors that may affect antibacterial activity.

By comparing the laboratory results, we verify the performance of 10 models (Supporting Information S12). The traditional machine‐learning models KNN, LGBM, RF and the deep learning models CNN and LSTM have an accuracy of 0.9. LGBM and LSTM correctly predicted the negative activity of Mag2(i + 4)15Dc but produced false negative results for Mag2(i + 4)14Dc and Mag2(i + 4)17Dc, respectively.

### 3.5 Haemolytic Activity of the Designed Stapled AMPs

Sheep red blood cells were used to investigate the haemolytic activity of the AMPs. As shown in Figure 7K, among the designed cysteine‐stapled peptides, both Mag2(i + 4)14Dc and Mag2(i + 4)17Dc exhibited haemolytic activity of more than 2.5% at a concentration of 256 μg/mL, whereas the haemolytic activities of Mag(i + 7)11St at 256 and 128 μg/mL were 29% and 24%, respectively, showing very strong haemolytic activity. For the designed lysine‐stapled peptides, the haemolytic activity at a concentration of 256 μg/mL was less than 2.5% in all cases. The above experimental results show that the designed lysine‐stapled peptides and cysteine‐stapled peptides performed well in terms of safety except for Mag(i + 7)11St.

### 3.6 Stability of the Designed Stapled AMPs

To further investigate the stability of our designed staple AMPs, we tested three cysteine‐stapled peptides and five lysine‐stapled peptides with high antimicrobial activity after incubation in 25% serum. As shown in Figure 7L, the residual antimicrobial activities of Mag2 and Mag2(i + 4)17Dc significantly decreased, whereas that of Mag(i + 4)0St decreased by approximately 50%, and that of Mag(i + 7)11St remained unchanged. Mag2(i + 4)17Dc was less stable in serum, whereas Mag(i + 4)0St and Mag(i + 7)11St were more stable than Mag2, indicating that hydrocarbon modification improved AMP stability. The residual antimicrobial activity of lysine‐stapled peptides neither improved nor decreased compared to that of their linear peptides, except for GAN‐P2‐3‐St, the stability of which decreased by approximately 25%. This may indicate that the stability of lysine‐stapled peptides is lower than that of cysteine‐stapled peptides.

### 3.7 CD Spectra of Linear Peptides and Stapled Peptides

**FIGURE 8:**
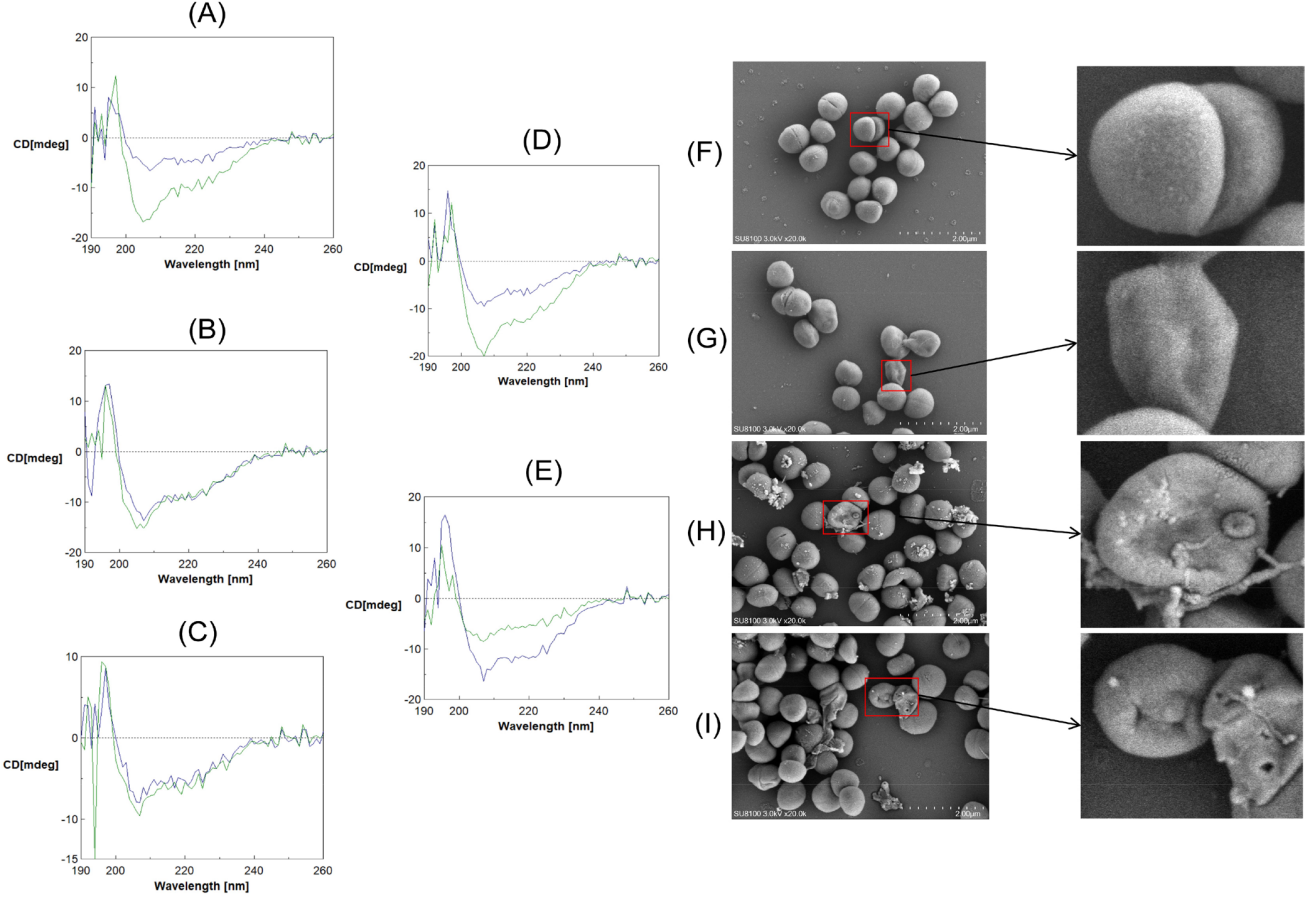
CD spectra of peptides in 100 mM SDS solution (linear peptides in blue, stapled peptides in green), (A) GAN‐P2‐2 (α‐helix = 58%) vs. GAN‐P2‐2‐St (α‐helix = 75%), (B) GAN‐P2‐3 (α‐helix = 75%) vs. GAN‐P2‐3‐St (α‐helix = 75%), (C) GAN‐P2‐4 (α‐helix = 35%) vs. GAN‐P2‐4‐St (α‐helix = 34%), (D) VAE‐P3‐3 (α‐helix = 35%) vs. VAE‐P3‐3‐St (α‐helix = 80%), (E) VAE‐P3‐5 (α‐helix = 76%) vs. VAE‐P3‐5‐St (α‐helix = 35%). SEM results of *S. aureus* incubated with (F) control, (G) 16 μg/mL GAN‐P2‐2, (H) 16 μg/mL GAN‐P2‐2‐St, (I) 32 μg/mL GAN‐P2‐2‐St.

CD spectra were obtained for linear and lysine‐stapled peptides. Both the linear and stapled peptides exhibited irregular coiling in PBS (Supporting Information S13), and both were α‐helix in 100 mM SDS solution (Figure 8A–E). Following the introduction of stapled structures, the α‐helix content of GAN‐P2‐2‐St and VAE‐P3‐3‐St increased, while that of GAN‐P2‐3‐St and GAN‐P2‐4‐St remained consistent with that of their linear peptides. Additionally, the antimicrobial activities of the four stapled peptides were observed to be elevated relative to their linear counterparts. This result indicates that the α‐helix content is not the sole determining factor of antimicrobial activity. Furthermore, it is notable that VAE‐P3‐5‐St exhibited a marked reduction in α‐helix content in comparison to its linear peptide, and its antimicrobial activity was also inferior to that of its linear peptide. This suggests that the incorporation of the stapled structure may also disrupt the secondary structure.

### 3.8 SEM Assay

Morphological alterations in *S. aureus* following incubation with the antimicrobial peptide were observed under an SEM. Incubation of *S. aureus* with the linear peptide at a concentration of 16 μg/mL resulted in slight deformation (Figure 8G). *S. aureus* incubated with the stapled peptide exhibited partial leakage of contents while undergoing deformation(Figure 8H). The structural collapse of *S. aureus* under incubation with a high concentration (32 μg/mL) of stapled peptide was accompanied by the formation of pores on the surface(Figure 8I). The observation of the shape change of *S. aureus* indicated that the antimicrobial peptide acted on the cell surface and that the antimicrobial properties of the stapled antimicrobial peptide were greater than those of the linear antimicrobial peptide.

## 4 Conclusion

To accelerate the design of stapled AMPs, we developed traditional machine and deep learning‐based models to predict the activity of stapled AMPs. These models combined 3D structural features with sequence and physicochemical features. The results showed that the isoelectric point (pro_pI_seq) had the greatest impact on the model's antimicrobial activity prediction. We then trained and compared the accuracies of seven traditional machine learning models and three deep learning models. The accuracy of these models was verified externally using an independent data set and by wet lab experiments. Five cysteine‐stapled AMPs derived from magainin II and five lysine‐stapled AMPs were designed and selected as positive candidates after screening using prediction models. They were synthesised and tested for their antibacterial activities against *E. coli* , *K. pneumoniae* , *S. aureus* and *P. aeruginosa* . The results showed that the four cysteine‐stapled peptides and five lysine‐stapled peptides had antibacterial activities comparable to those of their linear peptides in vitro. Moreover, lysine‐stapled peptides showed good antimicrobial activity against *MRSA*. The haemolytic activity indicates that, except for Mag (i + 7) 11St, most stapled peptides showed acceptable haemolytic activity. The secondary structures of stapled peptides and linear peptides were analysed using CD spectra, and the results demonstrated that the impact of stapled peptides on the secondary structure is inconclusive. Furthermore, it was established that this effect is not the sole factor influencing the antibacterial activity of these peptides. The effects of antimicrobial peptides on bacteria were observed by SEM, which revealed that antimicrobial peptides primarily exerted their bactericidal effects by destroying bacterial cell membranes. Furthermore, the destructive power of stapled peptides on cell membranes was found to exceed that of linear antimicrobial peptides. In summary, the deep‐learning models CNN and LSTM demonstrated high accuracy in predicting staple AMPs in both external independent datasets and our wet laboratory experiments. This indicates that deep learning has the potential to guide the rational design of antimicrobial peptides.

In recent years, stapled peptides have attracted widespread attention and have been used as protein–protein interaction (PPI) simulators, anticancer peptides and antimicrobial peptides because they can stabilise the secondary structure of peptides. However, stapled structures may lead to substantial alterations in activity, cell specificity, and stability (Migoń et al. 2018). Breakthroughs in the synthesis of stapled peptides have accelerated the screening of positive candidates. In a study by Mourtada et al. (2019), the synthesis of staple linkages was catalysed by dichloroethane and ruthenium, which are expensive and may introduce toxic by‐products. The mode of synthesis of cysteine‐stapled peptides used in our study is more promising as it uses NMP as a solvent, and the reaction can be performed at 365 nm, which is efficient and cheaper (Wang and Chou 2015). Serum stability experiments showed that cysteine hydrocarbon‐stapled modifications can significantly improve serum stability. However, there was still a significant increase in the haemolytic activity. Therefore, we utilised lysine stapling, a different method of stapled modification, and the results showed that it could be effective in controlling haemolytic activity. Nevertheless, the introduction of mutations and alterations into stapled peptides has the potential to yield a substantial array of novel stapled peptides, thereby increasing the demand for expeditious designs and screening methodologies.

Artificial intelligence (AI) combined algorithms, including machine learning and big data analytics, have emerged as a potential solution for analysing diverse data sets to identify biomolecules for effective treatment and prevention of infectious diseases (Al Meslamani et al. 2024). Most current AMP prediction methods are based on the primary sequence of AMPs, and the commonly used features are the amino acid sequence, amino acid composition, physical properties and others (Yan et al. 2023; Dean and Walper 2020). For example, Das et al. (2021) used deep learning classifiers combined with molecular dynamics to screen and obtain YI12 and FK13 with high in vitro antimicrobial activity. Mao et al. (2022) presented a deep‐generative network‐based approach for the design of AMPs, named AMPTrans‐lstm, which connected the LSTM sampler and transformer converter to generate novel and diverse AMPs. Although these feature extraction methods performed well on natural amino acid peptides, they cannot describe the structural features of bridge rings in staple peptides. For a long time, it has been difficult to include structural features in machine‐learning models because of the lack of experimentally determined peptide structures. The development of AI‐based structure prediction methods, such as AlphaFold, makes it possible to accurately and quickly model peptide structures. As McDonald's research (McDonald et al. 2023) points out, AlphaFold can predict α‐helical, β‐hairpin and disulfide‐rich peptides with high accuracy. In our study, the main peptide chain structure was predicted using AlphaFold and Swiss‐model, and the hydrocarbon linkage of the stapled peptide was added and minimised using MOE software. Subsequently, 3D structure descriptors were computed and used for model construction. We also comprehensively compared the performances of widely used traditional machine learning and deep learning methods. In the 10‐fold cross‐validation test, all 10 models demonstrated acceptable accuracy. The ACC values ranged from 0.719 to 0.884, and the AUC values ranged from 0.778 to 0.912. The SVM model performed the best, achieving the highest scores for four of the six indicators. Two of the deep‐learning models, CNN and LSTM, outperformed all the traditional machine learning models when validated using the external independent test and in our wet laboratory experiment, demonstrating that a deep learning‐based model is a rational approach to guide the design of stapled AMPs. In previous studies, the antimicrobial activity of stapled antimicrobial peptides was screened by synthesising and measuring the peptides. The synthesis cycle of stapled peptides is typically twice as long as that of linear peptides, and the initial screening of stapled antimicrobial peptides is expected to significantly reduce the time and cost of stapled antimicrobial peptide research.

Our study demonstrates that the isoelectric point and log octanol/water partition coefficient (SLopP) of stapled peptides are significantly associated with their antimicrobial activity. As SLogP is commonly used to measure the hydrophobicity of compounds, the observation is analogous to the outcomes in the case of linear antimicrobial peptides, wherein the features that have been identified as significant in AI4AMP encompass hydrophobicity and isoelectric point. The antibacterial activities of AMPs are highly correlated with the stabilities of their secondary structures (Cao et al. 2023), hydrophobicity values (Boopathi et al. 2019) and amphiphilicity values (Timmons and Hewage 2021). The stability of the peptides' secondary structure was confirmed to have a limited effect on their antimicrobial activity through the analysis of CD. In our study, the isoelectric point (pro_pI_seq) was the most significant factor influencing antimicrobial activity. This difference may be attributed to structural differences between the stapled and linear peptides. Thus, more consideration should be given to the influence of electrical properties on their activity during the design of stapled AMPs.

The type and position of the staple have a significant impact on the activity of stapled AMPs (Mourtada et al. 2019; Li et al. 2020). Because of the structural diversity of stapled rings, developing a common prediction model for stapled peptides is challenging. This study dichotomised the stapled peptides based on their antimicrobial activity, yet did not provide a detailed distinction between the strength of this activity. It is important to note that antimicrobial peptides exhibit varying antimicrobial activities against different bacteria, which may have contributed to the discrepancy between the evaluation and validation results. Furthermore, the experimental findings indicate that augmenting the stapled structure engenders an enhancement in activity, accompanied by a potential escalation in haemolysis. Consequently, it is imperative to select linear antimicrobial peptides with reduced haemolytic toxicity during the design process. The construction of a prediction model for haemolytic toxicity of stapled peptides has the potential to enhance the efficacy of the design process.

It was observed that the predictive models displayed a level of difference in their accuracy, which may be due to the relatively limited data used for training in the experiments. Deep learning models seem to have shown better performance compared to traditional machine learning algorithms. However, due to the limited number of negative samples, this may limit our observations of the predictive model's ability to distinguish false positives. We will consider adding more negative data to validate the model performance in subsequent studies. Furthermore, there are cases where certain peptides, such as Mag2(i + 4)15Dc in this study, have been experimentally classified as negative, even though the deep learning model predicted them to be positive. Additional algorithmic enhancements are necessary for small sample datasets, or using data sample expansion techniques to increase the amount of data used for training, which will improve prediction accuracy. In this study, only 181 hydrocarbon‐stapled AMPs were obtained from the DRAMP (Shi et al. 2021) database, which affected the accuracy of the prediction model. The sample sizes of positive data for AmPEP (Bhadra et al. 2018) and AMPpred (Meher et al. 2017) were 3268 and 3417, respectively. Further research on stapled AMPs will provide additional data to improve the accuracy of deep learning, accelerating the field of stapled AMPs (Cao et al. 2023; Cong and Zhou 2022).

Through model screening, 10 stapled peptides were synthesised. The designed lysine‐stapled peptides demonstrated good antibacterial activity against both *K. pneumoniae* and *S. aureus* , as well as against the drug‐resistant bacterium *MRSA*; the haemolytic activities were all less than 5% at 256 μg/mL. Experiments have shown that the five lysine‐stapled peptides have high antimicrobial activity and low haemolytic activity, indicating the potential for development and that lysine stapling is a reasonable strategy for AMP modification.

## Author Contributions

**Ruole Chen:** conceptualization, methodology, data curation, visualization, writing – review and editing, writing – original draft, validation, investigation, formal analysis. **Yuhao You:** conceptualization, methodology, validation, investigation, software, data curation, visualization, writing – review and editing. **Yanchao Liu:** visualization, data curation, resources. **Xin Sun:** data curation, resources, visualization. **Tianyue Ma:** investigation, validation, software, data curation. **Xingzhen Lao:** supervision, funding acquisition, writing – review and editing. **Heng Zheng:** conceptualization, methodology, writing – review and editing, writing – original draft, supervision, resources, project administration, formal analysis.

## Conflicts of Interest

The authors declare no conflicts of interest.

## Supporting information


Supporting Information S1.
**Supporting Information S2**.
**Supporting Information S3**.
**Supporting Information S5**.
**Supporting Information S6**.
**Supporting Information S7**.
**Supporting Information S8**.
**Supporting Information S9**.
**Supporting Information S10**.
**Supporting Information S11**.
**Supporting Information S12**.

Supporting Information S4.

## Data Availability

The code is available at GitHub (https://github.com/NancyBlack6/StAMPs).
