# Supplementary material for: Deep‐Learning‐Based Approaches for Rational Design of Stapled Peptides With High Antimicrobial Activity and Stability
Source: Microb Biotechnol. 2025 Mar 5;18(3):e70121. doi: 10.1111/1751-7915.70121 (PMC11881016; doi:10.1111/1751-7915.70121)

**Supplement S1** Intersection of output features of wrapper package method and sequential selection method

| Descriptor Meaning | Descriptor |
| --- | --- |
| Atomic counting and bond counting | a_ICM |
| Adjacency and distance matrix | BCUT_PEOE_3 |
|  | BCUT_SMR_0 |
|  | BCUT_SMR_3 |
|  | GCUT_PEOE_0 |
|  | GCUT_PEOE_2 |
|  | GCUT_PEOE_3 |
|  | GCUT_SLOGP_3 |
|  | GCUT_SMR_1 |
| dipole moment | dipole |
| Potential energy descriptor | E |
|  | E_sol |
|  | E_vdw |
| Shocker Theory Descriptor | h_pKa |
| Surface area, volume, and shape | npr2 |
|  | pmiX |
|  | std_dim2 |
|  | vsurf_CW6 |
|  | vsurf_D8 |
|  | vsurf_DD12 |
|  | vsurf_EWmin2 |
|  | vsurf_ID2 |
|  | vsurf_ID4 |
|  | vsurf_ID7 |
|  | vsurf_ID8 |
|  | vsurf_IW2 |
|  | vsurf_IW8 |
| Charge descriptor | PEOE_VSA_FPPOS |
| Protein patch descriptor | pro_patch_hyd_2 |
|  | pro_pI_3D |
|  | pro_pI_seq |
| physical property | rsynth |

**Supplement S2** Types of features obtained from K-means cluster analysis： K-means-1、K-means-2、K-means-3

| K-means-1 | K-means-2 | K-means-3 | | | |
| --- | --- | --- | --- | --- | --- |
| DASA | ASA_H | apol | chiral | PEOE_VSA-6 | vdw_area |
| FASA+ | b_1rotN | ASA | chiral_u | PEOE_VSA_HYD | vdw_vol |
| FCharge | b_rotN | ASA+ | DCASA | PEOE_VSA_NEG | vol |
| GCUT_PEOE_2 | diameter | ASA- | E_oop | PEOE_VSA_PNEG | VSA |
| h_logS | dipole | ASA_P | E_tor | PEOE_VSA_POL | vsa_acc |
| h_pavgQ | PEOE_VSA-1 | a_acc | FCASA+ | PEOE_VSA_POS | vsa_acid |
| PEOE_VSA+6 | pmi | a_acid | FCASA- | PEOE_VSA_PPOS | vsa_don |
| pro_app_charge | pmi2 | a_count | h_ema | pmi1 | vsa_hyd |
| pro_mobility | pmi3 | a_don | h_emd | pro_asa_vdw | vsa_other |
| pro_net_charge | pmiX | a_donacc | h_emd_C | pro_coeff_fric | vsa_pol |
| pro_patch_ion | pmiY | a_heavy | h_log_pbo | pro_mass | vsurf_D8 |
| pro_patch_ion_1 | pmiZ | a_hyd | h_mr | pro_r_solv | vsurf_HB1 |
| pro_patch_ion_2 | pro_asa_hyd | a_IC | Kier1 | pro_sed_const | vsurf_HB2 |
| pro_patch_ion_3 | pro_dipole_moment | a_nC | Kier2 | pro_volume | vsurf_HB3 |
| pro_patch_ion_4 | pro_hyd_moment | a_nH | Kier3 | Q_PC+ | vsurf_HB4 |
| pro_patch_ion_5 | pro_patch_hyd | a_nN | KierA1 | Q_VSA_HYD | vsurf_HB5 |
| pro_patch_ion_n | pro_patch_hyd_2 | a_nO | KierA2 | Q_VSA_NEG | vsurf_HB6 |
| pro_patch_pos | pro_patch_hyd_3 | bpol | KierA3 | Q_VSA_PNEG | vsurf_HB7 |
| pro_patch_pos_1 | pro_patch_hyd_4 | b_count | KierFlex | Q_VSA_POL | vsurf_S |
| pro_patch_pos_2 | pro_patch_hyd_5 | b_double | lip_acc | Q_VSA_POS | vsurf_V |
| pro_patch_pos_3 | pro_patch_hyd_n | b_heavy | lip_don | Q_VSA_PPOS | vsurf_W1 |
| pro_patch_pos_4 | pro_r_gyr | b_single | mr | SlogP_VSA0 | vsurf_W2 |
| pro_patch_pos_5 | rgyr | CASA+ | opr_nrot | SlogP_VSA1 | vsurf_W3 |
| pro_patch_pos_n | SlogP_VSA4 | CASA- | PC+ | SlogP_VSA2 | vsurf_W4 |
| pro_pI_3D | SlogP_VSA9 | chi0 | PEOE_PC+ | SlogP_VSA3 | vsurf_W5 |
| pro_pI_seq | SMR_VSA7 | chi0v | PEOE_VSA+0 | SlogP_VSA8 | vsurf_W6 |
| pro_zdipole | std_dim1 | chi0v_C | PEOE_VSA+1 | SMR | vsurf_W7 |
| pro_zeta | VDistEq | chi0_C | PEOE_VSA+2 | SMR_VSA0 | vsurf_Wp1 |
| pro_zquadrupole | vsurf_D1 | chi1 | PEOE_VSA+3 | SMR_VSA2 | vsurf_Wp2 |
| vsurf_IW1 | vsurf_D2 | chi1v | PEOE_VSA+4 | SMR_VSA3 | vsurf_Wp3 |
|  | vsurf_D3 | chi1v_C | PEOE_VSA-0 | SMR_VSA5 | Weight |
|  | vsurf_D4 | chi1_C | PEOE_VSA-5 | SMR_VSA6 | weinerPath |
|  | vsurf_D5 | TPSA | VAdjMa | VDistMa | weinerPol |
|  | vsurf_D6 |  |  |  | zagreb |
|  | vsurf_D7 |  |  |  |  |
|  | vsurf_G |  |  |  |  |

**Supplement S3** Types of features obtained from K-means cluster analysis： K-means-4、K-means-5、K-means-6

| K-means-4 | K-means-5 | | K-means-6 | |
| --- | --- | --- | --- | --- |
| BCUT_PEOE_1 | a_aro | pro_coeff_280 | ast_violation | petitjean |
| BCUT_SLOGP_1 | a_base | pro_eccen | ast_violation_ext | petitjeanSC |
| E_ele | a_ICM | Q_VSA_FPOL | a_nCl | pro_debye |
| FASA_H | a_nS | Q_VSA_FPPOS | balabanJ | pro_helicity |
| GCUT_PEOE_1 | BCUT_PEOE_2 | rings | BCUT_PEOE_0 | pro_henry |
| GCUT_SLOGP_1 | BCUT_SLOGP_2 | rsynth | BCUT_PEOE_3 | pro_patch_hyd_1 |
| GCUT_SMR_1 | BCUT_SMR_2 | SlogP_VSA5 | BCUT_SLOGP_0 | pro_patch_neg |
| h_logD | b_ar | SlogP_VSA7 | BCUT_SLOGP_3 | pro_patch_neg_1 |
| h_logP | dens | SMR_VSA1 | BCUT_SMR_0 | pro_patch_neg_2 |
| h_pKb | density | SMR_VSA4 | BCUT_SMR_1 | pro_patch_neg_3 |
| logP(o/w) | E_ang | std_dim2 | BCUT_SMR_3 | pro_patch_neg_4 |
| logS | FASA_P | std_dim3 | b_1rotR | pro_patch_neg_5 |
| PC- | GCUT_PEOE_3 | vsa_base | b_max1len | pro_patch_neg_n |
| PEOE_PC- | GCUT_SLOGP_2 | vsurf_CW2 | b_rotR | Q_VSA_FNEG |
| PEOE_RPC+ | GCUT_SLOGP_3 | vsurf_CW3 | b_triple | Q_VSA_FPNEG |
| PEOE_RPC- | GCUT_SMR_2 | vsurf_CW4 | dipoleX | Q_VSA_FPOS |
| PEOE_VSA_FHYD | GCUT_SMR_3 | vsurf_CW5 | dipoleY | radius |
| pro_coeff_diff | glob | vsurf_CW6 | dipoleZ | reactive |
| Q_PC- | h_pstates | vsurf_CW7 | E | SlogP_VSA6 |
| Q_RPC+ | npr1 | vsurf_CW8 | E_nb | vsurf_A |
| Q_RPC- | opr_brigid | vsurf_HB8 | E_rele | vsurf_DD12 |
| Q_VSA_FHYD | opr_nring | vsurf_HL1 | E_rnb | vsurf_DD13 |
| RPC+ | opr_violation | vsurf_HL2 | E_rsol | vsurf_DD23 |
| RPC- | PEOE_VSA+5 | vsurf_R | E_rvdw | vsurf_DW12 |
| SlogP | PEOE_VSA-4 | vsurf_W8 | E_sol | vsurf_DW13 |
| VAdjEq | PEOE_VSA_FPNEG | vsurf_Wp4 | E_str | vsurf_DW23 |
| vsurf_CP | PEOE_VSA_FPOL | vsurf_Wp5 | E_strain | vsurf_ID1 |
| vsurf_CW1 | PEOE_VSA_FPOS | vsurf_Wp6 | E_vdw | vsurf_ID2 |
| vsurf_EDmin1 | PEOE_VSA_FPPOS | vsurf_Wp7 | FASA- | vsurf_ID3 |
| vsurf_EDmin2 | pro_asa_hph | vsurf_Wp8 | GCUT_PEOE_0 | vsurf_ID4 |
| vsurf_EDmin3 |  |  | GCUT_SLOGP_0 | vsurf_ID5 |
| vsurf_EWmin1 |  |  | GCUT_SMR_0 | vsurf_ID6 |
| vsurf_EWmin2 |  |  | h_pKa | vsurf_ID7 |
| vsurf_EWmin3 |  |  | h_pstrain | vsurf_ID8 |
|  |  |  | lip_violation | vsurf_IW2 |
|  |  |  | mutagenic | vsurf_IW3 |
|  |  |  | nmol | vsurf_IW4 |
|  |  |  | npr2 | vsurf_IW5 |
|  |  |  | PEOE_VSA-2 | vsurf_IW6 |
|  |  |  | PEOE_VSA-3 | vsurf_IW7 |
|  |  |  | PEOE_VSA_FNEG | vsurf_IW8 |


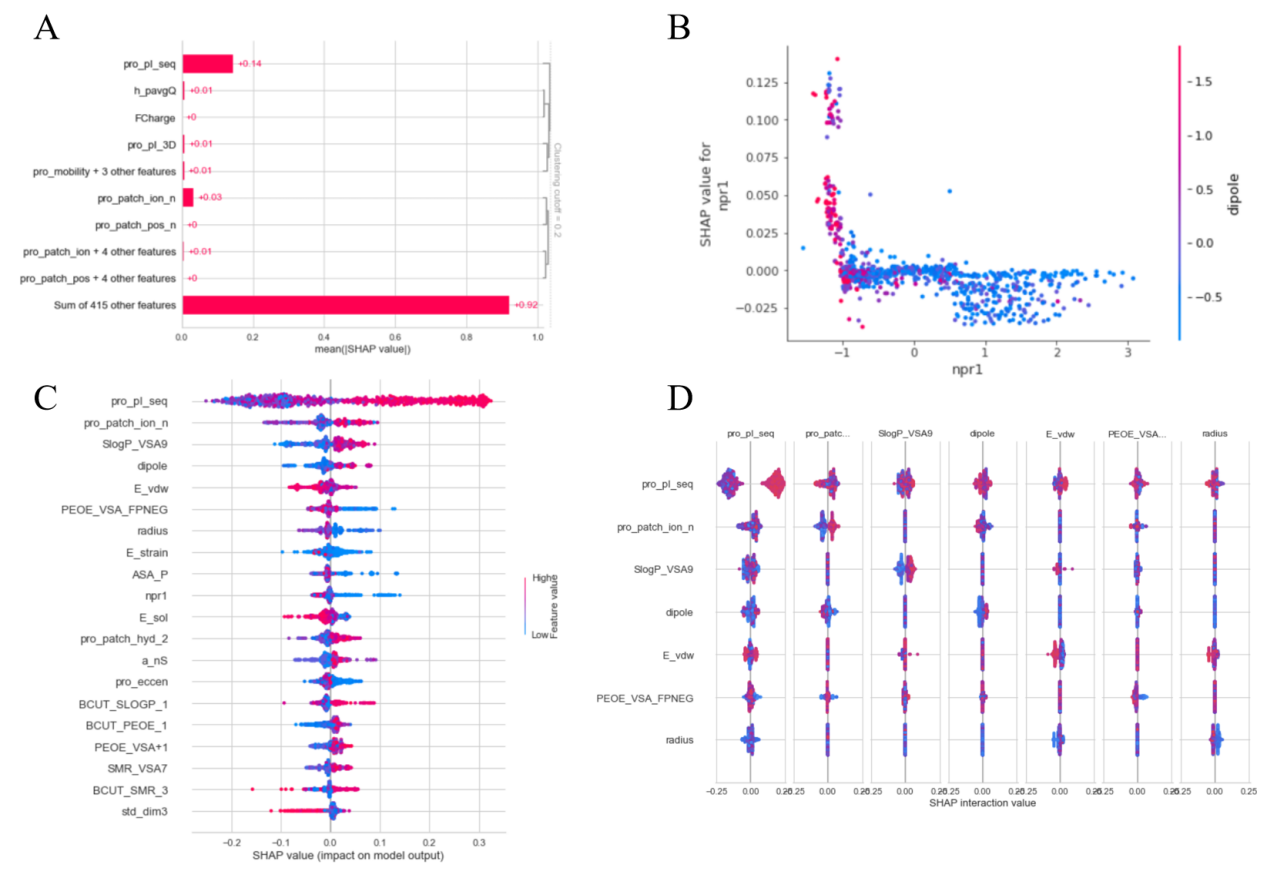


**Supplement S4.** Feature correlation summary generated by SHAP (A) Hierarchical clustering chart of features, reflecting the correlation among features. (B) Feature dependence scatter plot, reflecting the influence of feature dipole on feature npr1. The X axis is the range of eigenvalues of feature npr1, and the Y axis is the SHAP value of feature npr1, demonstrating the extent to which the feature modifies the model's output. (C) Beeswarm plot with SHAP value as X axis and descriptor feature as Y axis. The right color scale encodes the SHAP value (D) feature interaction plot.

**Supplement S5** Retained feature descriptors through filtering

| Number | Descriptor | Number | Descriptor |
| --- | --- | --- | --- |
| 1 | a_count | 55 | Q_VSA_FPPOS |
| 2 | a_hyd | 56 | SlogP_VSA8 |
| 3 | ASA | 57 | SlogP_VSA9 |
| 4 | ASA- | 58 | SMR_VSA5 |
| 5 | ASA_H | 59 | std_dim1 |
| 6 | ASA_P | 60 | std_dim2 |
| 7 | ASA+ | 61 | VDistEq |
| 8 | balabanJ | 62 | vsurf_A |
| 9 | BCUT_PEOE_0 | 63 | vsurf_D7 |
| 10 | BCUT_PEOE_1 | 64 | vsurf_D8 |
| 11 | BCUT_PEOE_2 | 65 | vsurf_EDmin1 |
| 12 | BCUT_PEOE_3 | 66 | vsurf_EDmin2 |
| 13 | BCUT_SLOGP_1 | 67 | vsurf_EDmin3 |
| 14 | CASA- | 68 | vsurf_EWmin1 |
| 15 | CASA+ | 69 | vsurf_EWmin2 |
| 16 | DASA | 70 | vsurf_EWmin3 |
| 17 | DCASA | 71 | vsurf_HL1 |
| 18 | dipole | 72 | vsurf_HL2 |
| 19 | dipoleX | 73 | vsurf_ID1 |
| 20 | E_ang | 74 | vsurf_ID2 |
| 21 | E_strain | 75 | vsurf_ID3 |
| 22 | E_vdw | 76 | vsurf_ID4 |
| 23 | GCUT_PEOE_0 | 77 | vsurf_ID5 |
| 24 | GCUT_PEOE_1 | 78 | vsurf_ID6 |
| 25 | GCUT_PEOE_3 | 79 | vsurf_ID7 |
| 26 | GCUT_SMR_0 | 80 | vsurf_ID8 |
| 27 | h_logD | 81 | vsurf_IW1 |
| 28 | h_pavgQ | 82 | vsurf_IW2 |
| 29 | h_pKa | 83 | vsurf_IW3 |
| 30 | h_pKb | 84 | vsurf_IW4 |
| 31 | h_pstrain | 85 | vsurf_IW5 |
| 32 | npr1 | 86 | vsurf_IW6 |
| 33 | npr2 | 87 | vsurf_IW7 |
| 34 | PEOE_PC- | 88 | vsurf_IW8 |
| 35 | PEOE_VSA_FHYD | 89 | vsurf_W1 |
| 36 | PEOE_VSA_FPNEG | 90 | vsurf_W3 |
| 37 | PEOE_VSA_FPPOS | 91 | vsurf_W5 |
| 38 | PEOE_VSA+2 | 92 | vsurf_W7 |
| 39 | PEOE_VSA+6 | 93 | BCUT_SMR_0 |
| 40 | pro_asa_hyd | 94 | BCUT_SMR_1 |
| 41 | pro_dipole_moment | 95 | BCUT_SMR_3 |
| 42 | pro_eccen | 96 | dipoleY |
| 43 | pro_hyd_moment | 97 | logS |
| 44 | pro_net_charge | 98 | pmi2 |
| 45 | pro_patch_hyd_1 | 99 | pmiX |
| 46 | pro_patch_hyd_2 | 100 | pmiY |
| 47 | pro_patch_ion_n | 101 | pmiZ |
| 48 | pro_patch_pos_4 | 102 | pro_helicity |
| 49 | pro_patch_pos_n | 103 | pro_sed_const |
| 50 | pro_pI_3D | 104 | pro_zdipole |
| 51 | pro_pI_seq | 105 | pro_zquadrupole |
| 52 | Q_PC- | 106 | vsurf_CW7 |
| 53 | Q_VSA_FHYD | 107 | vsurf_CW8 |
| 54 | Q_VSA_FPNEG |  |  |

**Supplement S6** Selection of each model parameter

In the case of the traditional machine learning model, the "GridSearchCV" function is called upon through the "model_selection" module in the "Scikit-learn" package with the objective of regulating the parameters. Once the range of hyperparameters has been selected by human beings, the code "GridSearchCV" is executed with the aim of identifying the optimal hyperparameter combinations through ten-fold cross-validation. The range of hyperparameters has been selected by the human operator, then the code "GridSearchCV" is executed to identify the optimal hyperparameter combination through ten-fold cross-validation. The area under the receiver operating characteristic curve (AUC) is employed as the evaluation metric to ascertain the most effective model.

The parameter tuning of the deep learning model is also performed by "GridSearchCV" in order to obtain the optimal parameters through the application of 10-fold cross-validation to the selected parameter ranges. Furthermore, deep learning models frequently undergo multiple training cycles. Consequently, the "ModelCheckpoint" and "EarlyStopping" callback functions are employed to monitor performance metrics. The "EarlyStopping" callback function is employed to monitor the performance metrics, thereby enabling the model to terminate the current training rounds and store the optimal model when further optimization is not possible.

| Model | Parameters |
| --- | --- |
| DT | criterion: gini、entropy  max_depth: 10-100 |
| KNN | n_neighbors(k): 1-50  weights: distance、algorithm、auto |
| LGBM | learning_rate: 0.05-0.1  n_estimators: 100-1000 |
| NB | default value |
| RF | n_estimators: 100-1200  max_features: auto  max_depth: 10-100  min_samples_split: 2-10  min_samples_leaf: 1-4 |
| SVM | kernel: linear、poly、rbf、sigmoid、precomputed  C: 1、10、100、1000  gamma: 0.01、0.001、0.0001、0.00001 |
| ANN | alpha: 0.1、0.01、0.001  hiden_layer_sizes: (10，10)、(20，20)、(50，50)、(100，100)、(10，10，10)、(20，20，20)、(50，50，50)、(100，100，100)  solver: lbfgs、adam、sgd  activation: logistic、tath、relu |
| CNN | filter: 1-14  kernel_size: (filter+1)*2  strides: 1  padding: same  activation: relu |
| LSTM | filter: 1-14  kernel_size: (filter+1)*2  hidden_size: 32-512  num_layers: 32-512  strides: 1  padding: same  activation: relu |

**Supplement S7** Designed disulfide-cyclic-cysteine-stapled peptides and the antimicrobial activity result according to the prediction model

| Peptide | Sequence | Machine learning | | | | | | | Deep learning | | |
| --- | --- | --- | --- | --- | --- | --- | --- | --- | --- | --- | --- |
|  |  | DT | KNN | LGBM | NB | SVM | RF | XGB | ANN | CNN | LSTM |
| Mag(i+4)0Dc | ©^a^IGK©LHSAKKFGKAFVGEILNS | 1.000 | 1.000 | 0.061 | 0.970 | 0.528 | 0.758 | 0.208 | 0.030 | 1.000 | 0.979 |
| Mag(i+4)1Dc | G©GKF©HSAKKFGKAFVGEILNS | 1.000 | 1.000 | 0.052 | 0.562 | 0.524 | 0.762 | 0.165 | 0.182 | 1.000 | 0.987 |
| Mag(i+4)2Dc | GI©KFL©SAKKFGKAFVGEILNS | 1.000 | 1.000 | 0.440 | 0.995 | 0.568 | 0.951 | 0.164 | 1.000 | 1.000 | 1.000 |
| Mag(i+4)3Dc | GIG©FLH©AKKFGKAFVGEILNS | 1.000 | 1.000 | 0.193 | 0.776 | 0.454 | 0.929 | 0.243 | 1.000 | 1.000 | 0.999 |
| Mag(i+4)4Dc | GIGK©LHS©KKFGKAFVGEILNS | 1.000 | 1.000 | 0.092 | 0.992 | 0.565 | 0.890 | 0.423 | 1.000 | 1.000 | 1.000 |
| Mag(i+4)5Dc | GIGKF©HSA©KFGKAFVGEILNS | 1.000 | 1.000 | 0.224 | 0.393 | 0.520 | 0.808 | 0.765 | 0.999 | 1.000 | 0.981 |
| Mag(i+4)6Dc | GIGKFL©SAK©FGKAFVGEILNS | 1.000 | 1.000 | 0.397 | 0.733 | 0.514 | 0.965 | 0.635 | 0.998 | 1.000 | 0.998 |
| Mag(i+4)7Dc | GIGKFLH©AKK©GKAFVGEILNS | 0.000 | 1.000 | 0.051 | 0.817 | 0.473 | 0.815 | 0.084 | 0.938 | 1.000 | 0.999 |
| Mag(i+4)8Dc | GIGKFLHS©KKF©KAFVGEILNS | 1.000 | 1.000 | 0.331 | 0.996 | 0.598 | 0.880 | 0.360 | 1.000 | 1.000 | 1.000 |
| Mag(i+4)9Dc | GIGKFLHSA©KFG©AFVGEILNS | 1.000 | 1.000 | 0.457 | 0.697 | 0.482 | 0.811 | 0.756 | 0.997 | 1.000 | 0.996 |
| Mag(i+4)10Dc | GIGKFLHSAK©FGK©FVGEILNS | 1.000 | 1.000 | 0.628 | 0.483 | 0.509 | 0.752 | 0.502 | 0.993 | 1.000 | 0.986 |
| Mag(i+4)11Dc | GIGKFLHSAKK©GKA©VGEILNS | 0.000 | 1.000 | 0.018 | 0.980 | 0.479 | 0.751 | 0.013 | 0.986 | 1.000 | 0.998 |
| Mag(i+4)12Dc | GIGKFLHSAKKF©KAF©GEILNS | 1.000 | 1.000 | 0.304 | 0.988 | 0.603 | 0.903 | 0.689 | 1.000 | 1.000 | 1.000 |
| Mag(i+4)13Dc | GIGKFLHSAKKFG©AFV©EILNS | 1.000 | 1.000 | 0.646 | 0.939 | 0.558 | 0.875 | 0.811 | 1.000 | 1.000 | 1.000 |
| Mag(i+4)14Dc | GIGKFLHSAKKFGK©FVG©ILNS | 1.000 | 1.000 | 0.195 | 0.978 | 0.571 | 0.627 | 0.218 | 0.997 | 1.000 | 0.999 |
| Mag(i+4)15Dc | GIGKFLHSAKKFGKA©VGE©LNS | 1.000 | 1.000 | 0.079 | 0.944 | 0.501 | 0.721 | 0.157 | 0.987 | 1.000 | 0.172 |
| Mag(i+4)16Dc | GIGKFLHSAKKFGKAF©GEI©NS | 1.000 | 1.000 | 0.135 | 0.847 | 0.552 | 0.854 | 0.337 | 0.851 | 1.000 | 0.702 |
| Mag(i+4)17Dc | GIGKFLHSAKKFGKAFV©EIL©S | 1.000 | 1.000 | 0.679 | 0.999 | 0.592 | 0.778 | 0.361 | 1.000 | 1.000 | 0.310 |
| Mag(i+4)18Dc | GIGKFLHSAKKFGKAFVG©ILN© | 1.000 | 1.000 | 0.521 | 0.992 | 0.573 | 0.755 | 0.110 | 0.998 | 1.000 | 0.007 |
| Mag(i+7)0Dc | ©IGKFLH©AKKFGKAFVGEILNS | 1.000 | 0.778 | 0.794 | 0.999 | 0.583 | 0.538 | 0.792 | 0.000 | 1.000 | 1.000 |
| Mag(i+7)1Dc | G©GKFLHS©KKFGKAFVGEILNS | 1.000 | 1.000 | 0.828 | 0.978 | 0.616 | 0.574 | 0.934 | 1.000 | 1.000 | 1.000 |
| Mag(i+7)2Dc | GI©KFLHSA©KFGKAFVGEILNS | 1.000 | 1.000 | 0.933 | 0.895 | 0.587 | 0.586 | 0.963 | 1.000 | 1.000 | 1.000 |
| Mag(i+7)3Dc | GIG©FLHSAK©FGKAFVGEILNS | 0.000 | 1.000 | 0.754 | 0.085 | 0.391 | 0.521 | 0.425 | 1.000 | 1.000 | 1.000 |
| Mag(i+7)4Dc | GIGK©LHSAKK©GKAFVGEILNS | 0.000 | 1.000 | 0.287 | 0.816 | 0.484 | 0.685 | 0.572 | 1.000 | 1.000 | 1.000 |
| Mag(i+7)5Dc | GIGKF©HSAKKF©KAFVGEILNS | 0.000 | 1.000 | 0.959 | 0.998 | 0.675 | 0.585 | 0.899 | 1.000 | 1.000 | 1.000 |
| Mag(i+7)6Dc | GIGKFL©SAKKFG©AFVGEILNS | 1.000 | 1.000 | 0.945 | 0.998 | 0.594 | 0.625 | 0.885 | 1.000 | 1.000 | 1.000 |
| Mag(i+7)7Dc | GIGKFLH©AKKFGK©FVGEILNS | 1.000 | 1.000 | 0.962 | 1.000 | 0.641 | 0.670 | 0.980 | 1.000 | 1.000 | 1.000 |
| Mag(i+7)8Dc | GIGKFLHS©KKFGKA©VGEILNS | 0.000 | 1.000 | 0.899 | 0.555 | 0.564 | 0.618 | 0.931 | 1.000 | 1.000 | 1.000 |
| Mag(i+7)9Dc | GIGKFLHSA©KFGKAF©GEILNS | 0.000 | 1.000 | 0.908 | 0.804 | 0.601 | 0.562 | 0.944 | 1.000 | 1.000 | 1.000 |
| Mag(i+7)10Dc | GIGKFLHSAK©FGKAFV©EILNS | 1.000 | 1.000 | 0.978 | 0.999 | 0.683 | 0.556 | 0.888 | 1.000 | 1.000 | 1.000 |
| Mag(i+7)11Dc | GIGKFLHSAKK©GKAFVG©ILNS | 0.000 | 0.556 | 0.844 | 0.987 | 0.601 | 0.647 | 0.917 | 1.000 | 1.000 | 1.000 |
| Mag(i+7)12Dc | GIGKFLHSAKKF©KAFVGE©LNS | 1.000 | 1.000 | 0.838 | 0.994 | 0.653 | 0.563 | 0.922 | 1.000 | 1.000 | 1.000 |
| Mag(i+7)13Dc | GIGKFLHSAKKFG©AFVGEI©NS | 1.000 | 1.000 | 0.930 | 0.983 | 0.602 | 0.572 | 0.979 | 1.000 | 1.000 | 1.000 |
| Mag(i+7)14Dc | GIGKFLHSAKKFGK©FVGEIL©S | 0.000 | 1.000 | 0.935 | 0.999 | 0.675 | 0.572 | 0.784 | 1.000 | 1.000 | 1.000 |
| Mag(i+7)15Dc | GIGKFLHSAKKFGKA©VGEILN© | 0.000 | 0.333 | 0.833 | 0.689 | 0.549 | 0.538 | 0.959 | 0.000 | 1.000 | 1.000 |

**a: cysteine that staple with others**

**Supplement S8** Designed hydrocarbon-cysteine-stapled peptides and the antimicrobial activity result according to the prediction model

| Peptide | Sequence | Machine learning | | | | | | | Deep learning | | |
| --- | --- | --- | --- | --- | --- | --- | --- | --- | --- | --- | --- |
|  |  | DT | KNN | LGBM | NB | SVM | RF | XGB | ANN | CNN | LSTM |
| Mag(i+4)0St | ©^a^IGK©LHSAKKFGKAFVGEIⓁNS | 1.000 | 1.000 | 0.762 | 1.000 | 0.647 | 0.971 | 0.495 | 1.000 | 1.000 | 1.000 |
| Mag(i+4)1St | G©GKF©HSAKKFGKAFVGEIⓁNS | 1.000 | 1.000 | 0.410 | 0.998 | 0.610 | 0.944 | 0.178 | 1.000 | 1.000 | 0.997 |
| Mag(i+4)2St | GI©KFL©SAKKFGKAFVGEIⓁNS | 0.000 | 1.000 | 0.269 | 1.000 | 0.622 | 0.965 | 0.400 | 1.000 | 1.000 | 0.999 |
| Mag(i+4)3St | GIG©FLH©AKKFGKAFVGEIⓁNS | 1.000 | 1.000 | 0.467 | 0.993 | 0.572 | 0.941 | 0.787 | 1.000 | 1.000 | 1.000 |
| Mag(i+4)4St | GIGK©LHS©KKFGKAFVGEIⓁNS | 1.000 | 1.000 | 0.671 | 1.000 | 0.648 | 0.937 | 0.478 | 1.000 | 1.000 | 0.999 |
| Mag(i+4)5St | GIGKF©HSA©KFGKAFVGEIⓁNS | 1.000 | 1.000 | 0.826 | 0.998 | 0.626 | 0.922 | 0.720 | 0.999 | 1.000 | 0.998 |
| Mag(i+4)6St | GIGKFL©SAK©FGKAFVGEIⓁNS | 1.000 | 1.000 | 0.679 | 0.992 | 0.578 | 0.964 | 0.545 | 1.000 | 1.000 | 0.999 |
| Mag(i+4)7St | GIGKFLH©AKK©GKAFVGEIⓁNS | 1.000 | 1.000 | 0.599 | 1.000 | 0.653 | 0.968 | 0.602 | 1.000 | 1.000 | 1.000 |
| Mag(i+4)8St | GIGKFLHS©KKF©KAFVGEIⓁNS | 1.000 | 1.000 | 0.507 | 1.000 | 0.680 | 0.939 | 0.106 | 1.000 | 1.000 | 1.000 |
| Mag(i+4)9St | GIGKFLHSA©KFG©AFVGEIⓁNS | 1.000 | 1.000 | 0.614 | 0.997 | 0.504 | 0.879 | 0.327 | 0.990 | 1.000 | 0.998 |
| Mag(i+4)10St | GIGKFLHSAK©FGK©FVGEIⓁNS | 1.000 | 1.000 | 0.601 | 0.990 | 0.584 | 0.765 | 0.640 | 0.981 | 1.000 | 0.971 |
| Mag(i+4)11St | GIGKFLHSAKK©GKA©VGEIⓁNS | 0.000 | 1.000 | 0.245 | 1.000 | 0.630 | 0.950 | 0.410 | 0.999 | 1.000 | 0.999 |
| Mag(i+4)12St | GIGKFLHSAKKF©KAF©GEIⓁNS | 1.000 | 1.000 | 0.645 | 1.000 | 0.664 | 0.929 | 0.452 | 1.000 | 1.000 | 0.999 |
| Mag(i+4)13St | GIGKFLHSAKKFG©AFV©EIⓁNS | 1.000 | 1.000 | 0.552 | 0.988 | 0.576 | 0.851 | 0.734 | 0.964 | 1.000 | 0.998 |
| Mag(i+4)14St | GIGKFLHSAKKFGK©FVG©IⓁNS | 1.000 | 1.000 | 0.289 | 0.999 | 0.629 | 0.831 | 0.059 | 0.998 | 1.000 | 0.999 |
| Mag(i+4)15St | GIGKFLHSAKKFGKA©VGE©ⓁNS | 1.000 | 1.000 | 0.750 | 1.000 | 0.602 | 0.807 | 0.411 | 0.401 | 1.000 | 0.029 |
| Mag(i+4)16St | GIGKFLHSAKKFGKAF©GEI©NS | 1.000 | 1.000 | 0.840 | 1.000 | 0.640 | 0.862 | 0.558 | 0.999 | 1.000 | 0.728 |
| Mag(i+4)17St | GIGKFLHSAKKFGKAFV©EIⓁ©S | 1.000 | 1.000 | 0.553 | 1.000 | 0.621 | 0.849 | 0.570 | 0.993 | 1.000 | 0.039 |
| Mag(i+4)18St | GIGKFLHSAKKFGKAFVG©IⓁN© | 0.000 | 1.000 | 0.785 | 1.000 | 0.632 | 0.928 | 0.291 | 1.000 | 1.000 | 0.168 |
| Mag(i+7)0St | ©IGKFLH©AKKFGKAFVGEIⓁNS | 1.000 | 0.667 | 0.780 | 1.000 | 0.583 | 0.538 | 0.830 | 0.003 | 1.000 | 1.000 |
| Mag(i+7)1St | G©GKFLHS©KKFGKAFVGEIⓁNS | 1.000 | 1.000 | 0.982 | 1.000 | 0.726 | 0.919 | 0.979 | 1.000 | 1.000 | 1.000 |
| Mag(i+7)2St | GI©KFLHSA©KFGKAFVGEIⓁNS | 0.000 | 1.000 | 0.976 | 1.000 | 0.711 | 0.979 | 0.959 | 1.000 | 1.000 | 1.000 |
| Mag(i+7)3St | GIG©FLHSAK©FGKAFVGEIⓁNS | 0.000 | 1.000 | 0.963 | 0.955 | 0.477 | 0.944 | 0.873 | 1.000 | 1.000 | 1.000 |
| Mag(i+7)4St | GIGK©LHSAKK©GKAFVGEIⓁNS | 1.000 | 1.000 | 0.959 | 1.000 | 0.689 | 0.941 | 0.945 | 1.000 | 1.000 | 1.000 |
| Mag(i+7)5St | GIGKF©HSAKKF©KAFVGEIⓁNS | 0.000 | 1.000 | 0.958 | 1.000 | 0.710 | 0.955 | 0.870 | 1.000 | 1.000 | 1.000 |
| Mag(i+7)6St | GIGKFL©SAKKFG©AFVGEIⓁNS | 1.000 | 1.000 | 0.982 | 1.000 | 0.662 | 0.959 | 0.978 | 1.000 | 1.000 | 1.000 |
| Mag(i+7)7St | GIGKFLH©AKKFGK©FVGEIⓁNS | 1.000 | 1.000 | 0.987 | 1.000 | 0.709 | 0.978 | 0.954 | 1.000 | 1.000 | 1.000 |
| Mag(i+7)8St | GIGKFLHS©KKFGKA©VGEIⓁNS | 0.000 | 1.000 | 0.963 | 1.000 | 0.732 | 0.968 | 0.950 | 1.000 | 1.000 | 1.000 |
| Mag(i+7)9St | GIGKFLHSA©KFGKAF©GEIⓁNS | 0.000 | 1.000 | 0.980 | 1.000 | 0.700 | 0.967 | 0.971 | 1.000 | 1.000 | 1.000 |
| Mag(i+7)10St | GIGKFLHSAK©FGKAFV©EIⓁNS | 1.000 | 1.000 | 0.981 | 0.994 | 0.669 | 0.971 | 0.911 | 1.000 | 1.000 | 1.000 |
| Mag(i+7)11St | GIGKFLHSAKK©GKAFVG©IⓁNS | 0.000 | 1.000 | 0.955 | 1.000 | 0.728 | 0.952 | 0.930 | 1.000 | 1.000 | 1.000 |
| Mag(i+7)12St | GIGKFLHSAKKF©KAFVGE©ⓁNS | 1.000 | 1.000 | 0.963 | 1.000 | 0.728 | 0.973 | 0.864 | 1.000 | 1.000 | 1.000 |
| Mag(i+7)13St | GIGKFLHSAKKFG©AFVGEI©NS | 1.000 | 1.000 | 0.983 | 1.000 | 0.678 | 0.947 | 0.841 | 1.000 | 1.000 | 1.000 |
| Mag(i+7)14St | GIGKFLHSAKKFGK©FVGEIⓁ©S | 0.000 | 1.000 | 0.976 | 1.000 | 0.683 | 0.940 | 0.812 | 1.000 | 1.000 | 1.000 |
| Mag(i+7)15St | GIGKFLHSAKKFGKA©VGEIⓁN© | 0.000 | 0.333 | 0.984 | 0.351 | 0.561 | 0.538 | 0.985 | 0.000 | 1.000 | 1.000 |

**a: cysteine that staple with others**

**Supplement S9** Designed lysine-stapled peptides and the antimicrobial activity result according to the prediction model

| Peptide | Sequence | Machine learning | | | | | | | Deep learning | | |
| --- | --- | --- | --- | --- | --- | --- | --- | --- | --- | --- | --- |
|  |  | DT | KNN | LGBM | NB | SVM | RF | XGB | ANN | CNN | LSTM |
| GAN-P2-St | RILRLKRFLK | 1.000 | 0.333 | 0.919 | 0.027 | 0.507 | 0.298 | 0.833 | 0.001 | 0.952 | 0.994 |
| GAN-P2-1-St | RILRKLKKFL | 0.000 | 0.889 | 0.983 | 0.688 | 0.602 | 0.268 | 0.929 | 0.143 | 0.997 | 1.000 |
| GAN-P2-2-St | RILRKLRKFL | 0.000 | 0.889 | 0.979 | 0.007 | 0.509 | 0.385 | 0.832 | 0.005 | 0.990 | 1.000 |
| GAN-P2-3-St | RILRKLKRFL | 0.000 | 0.889 | 0.986 | 0.569 | 0.522 | 0.381 | 0.907 | 0.198 | 0.985 | 1.000 |
| GAN-P2-4-St | RILRKLRRFL | 0.000 | 0.778 | 0.956 | 0.001 | 0.492 | 0.482 | 0.892 | 0.001 | 0.990 | 0.999 |
| VAE-P3-St | WRIARLKRKLGRAKLF | 0.000 | 1.000 | 0.783 | 0.999 | 0.662 | 0.332 | 0.518 | 0.941 | 1.000 | 0.999 |
| VAE-P3-1-St | WKRIARLKRLGRAKLF | 0.867 | 0.778 | 0.974 | 1.000 | 0.711 | 0.298 | 0.851 | 0.903 | 1.000 | 1.000 |
| VAE-P3-2-St | WKRIARLRKLGRAKLF | 0.867 | 0.889 | 0.939 | 1.000 | 0.713 | 0.365 | 0.573 | 0.430 | 1.000 | 1.000 |
| VAE-P3-3-St | WKRIARLKKLGRAKLF | 0.867 | 1.000 | 0.982 | 1.000 | 0.745 | 0.538 | 0.667 | 0.996 | 1.000 | 1.000 |
| VAE-P3-4-St | WKRIAKLKRLGRAKLF | 0.867 | 0.778 | 0.992 | 1.000 | 0.745 | 0.313 | 0.636 | 0.529 | 1.000 | 1.000 |
| VAE-P3-5-St | WKRIARLKRLGRAKLF | 0.867 | 1.000 | 0.989 | 1.000 | 0.727 | 0.565 | 0.514 | 0.940 | 1.000 | 1.000 |

**a: cysteine that staple with others**

**Supplement S10** Purity of synthetic staple peptides and linear peptides (HPLC)

GAN-P2-2:


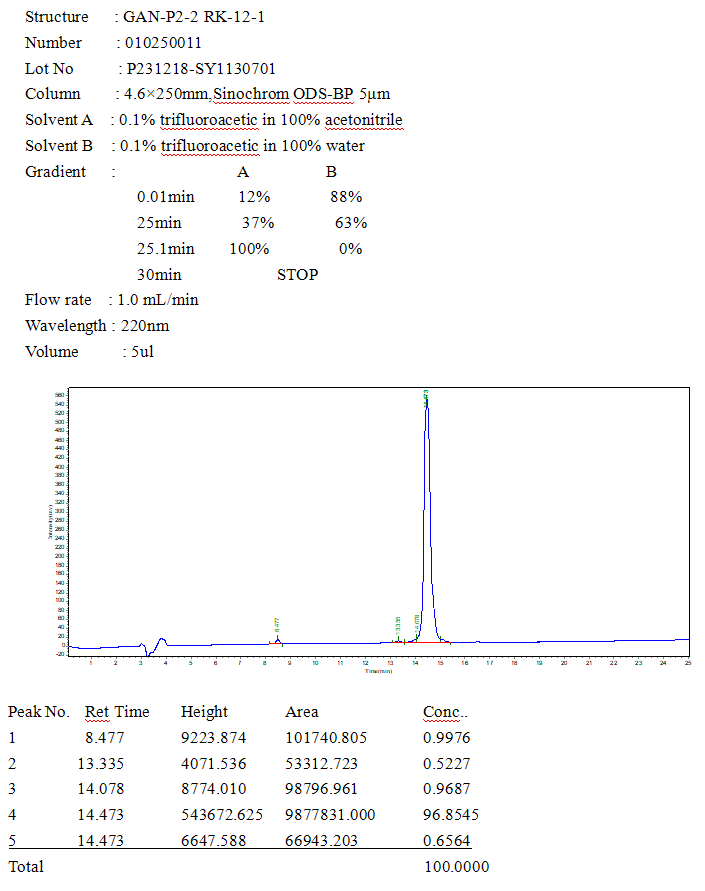


GAN-P2-2-Ly:


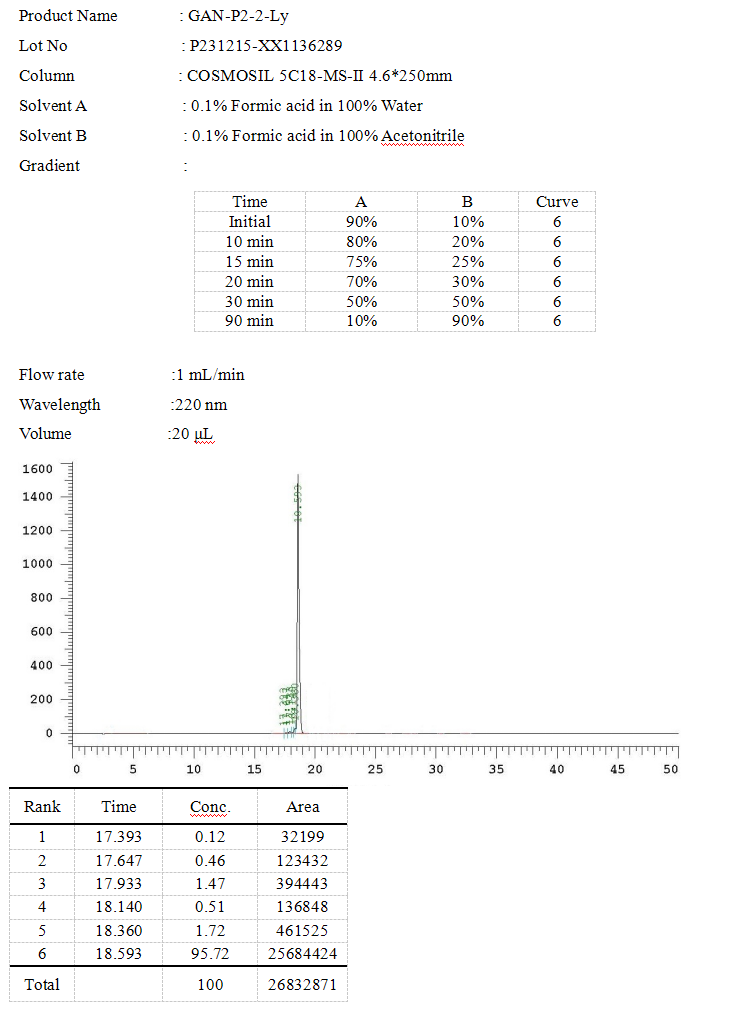


GAN-P2-3:


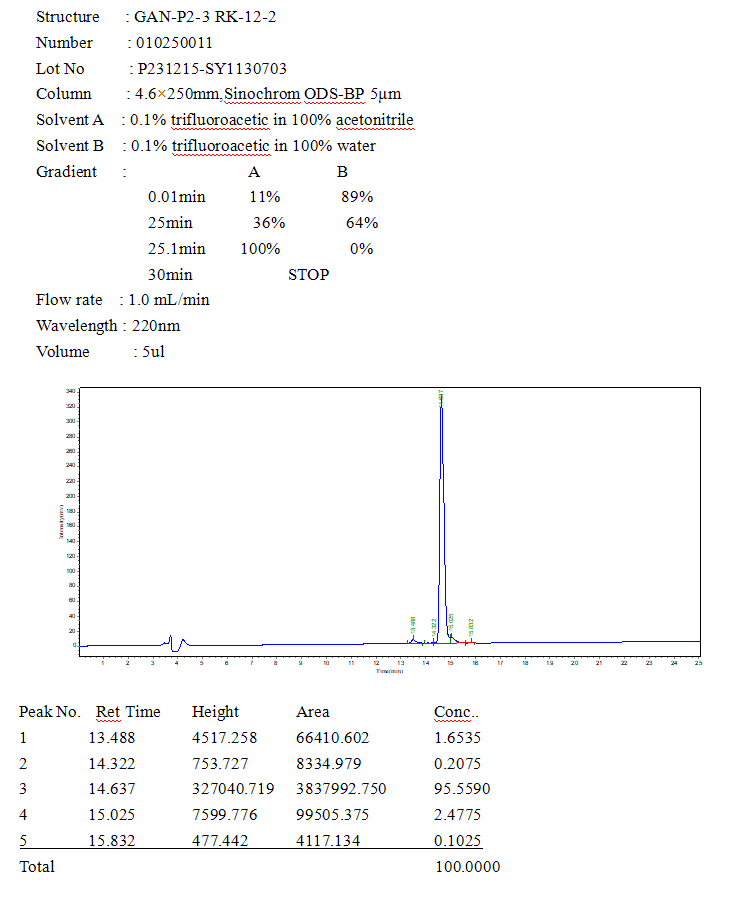


GAN-P2-3-Ly:


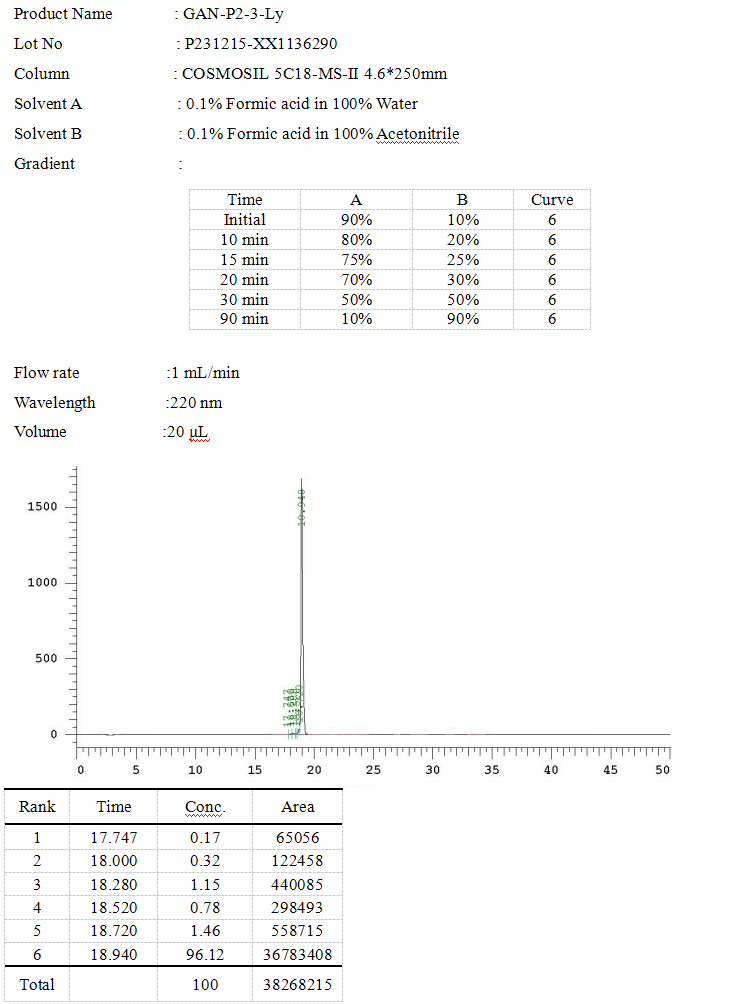


GAN-P2-4:


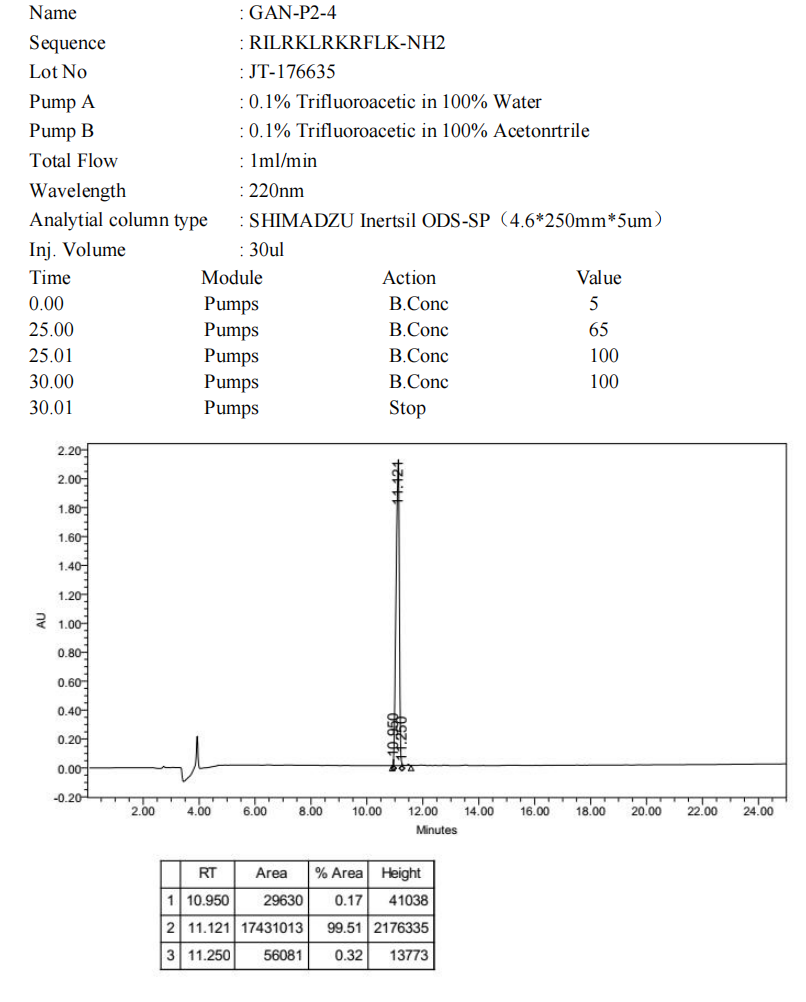


GAN-P2-4-Ly:


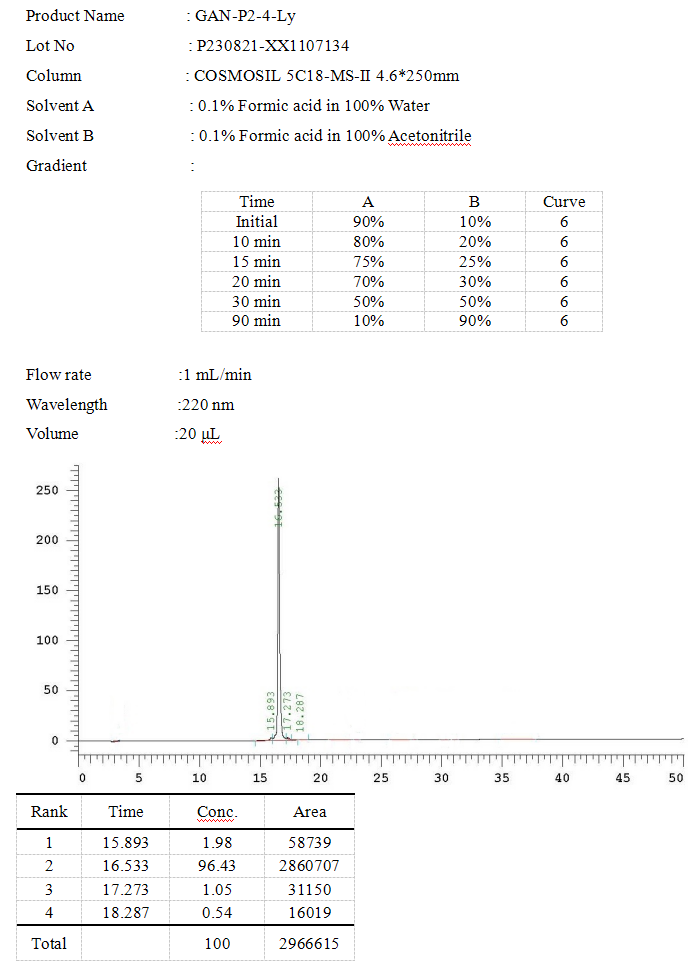


VAE-P3-3:


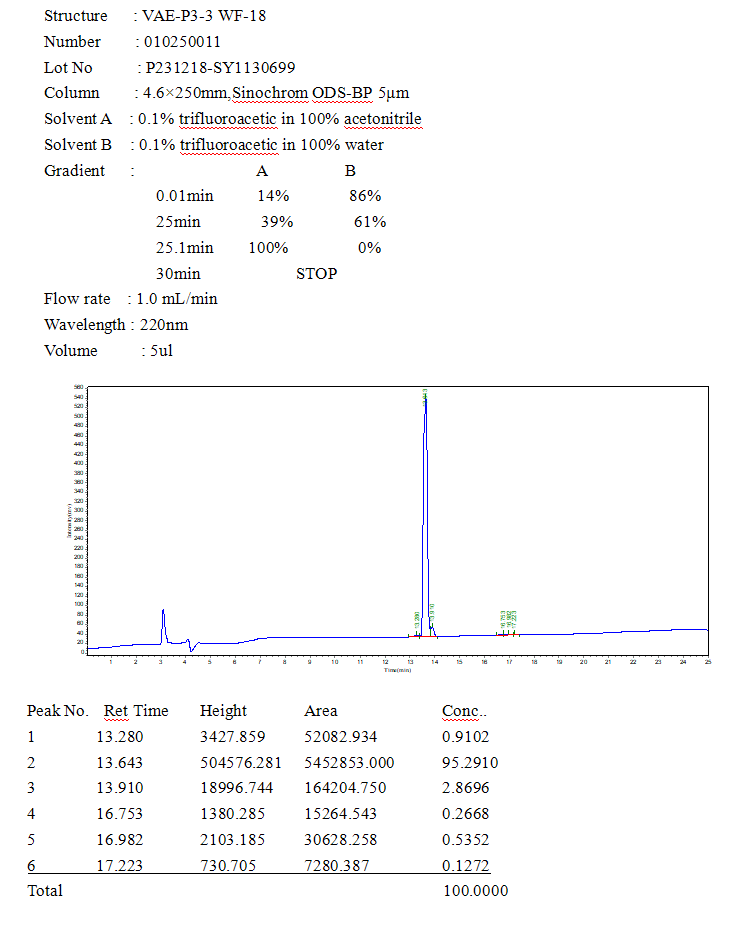


VAE-P3-3-Ly:


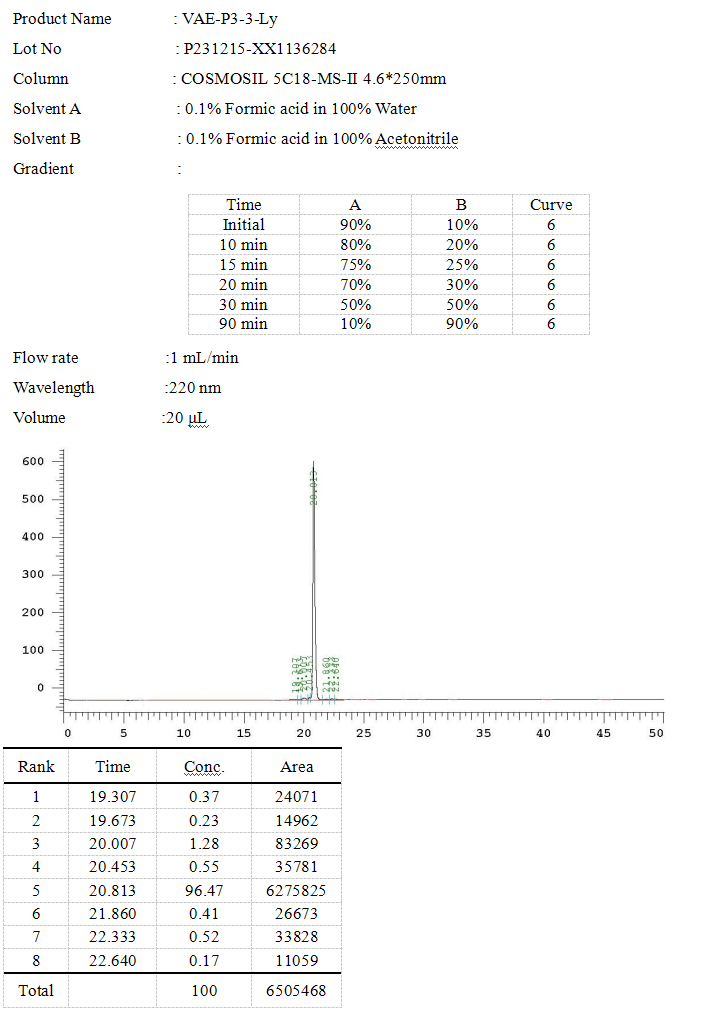


VAE-P3-5:


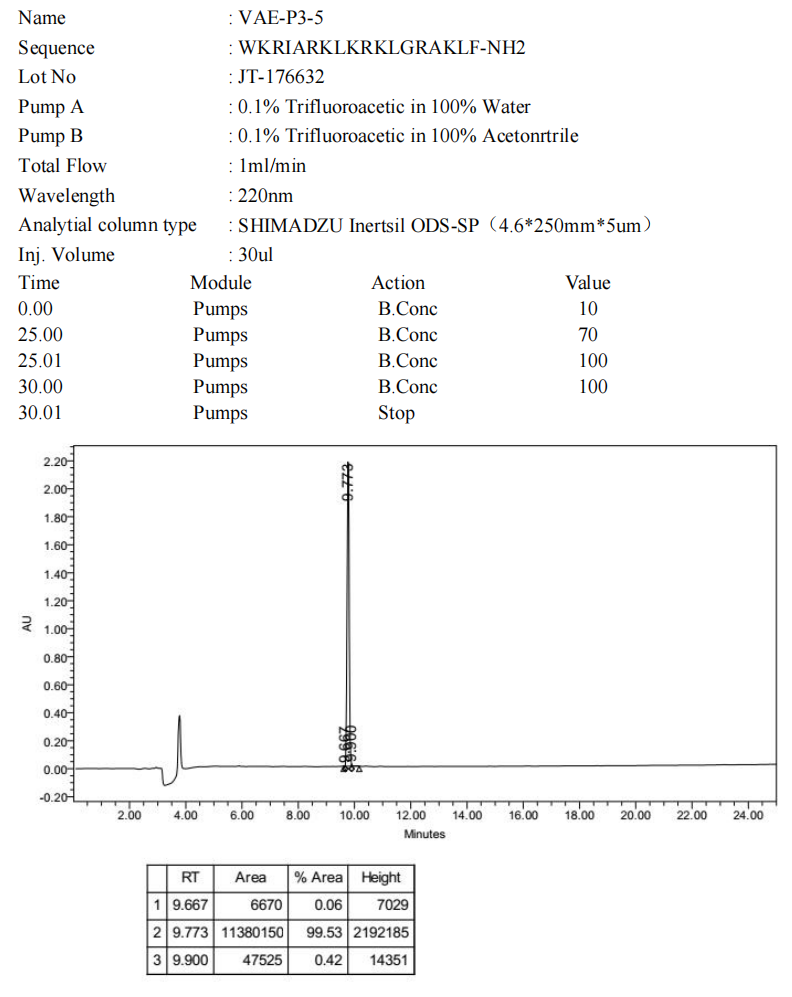


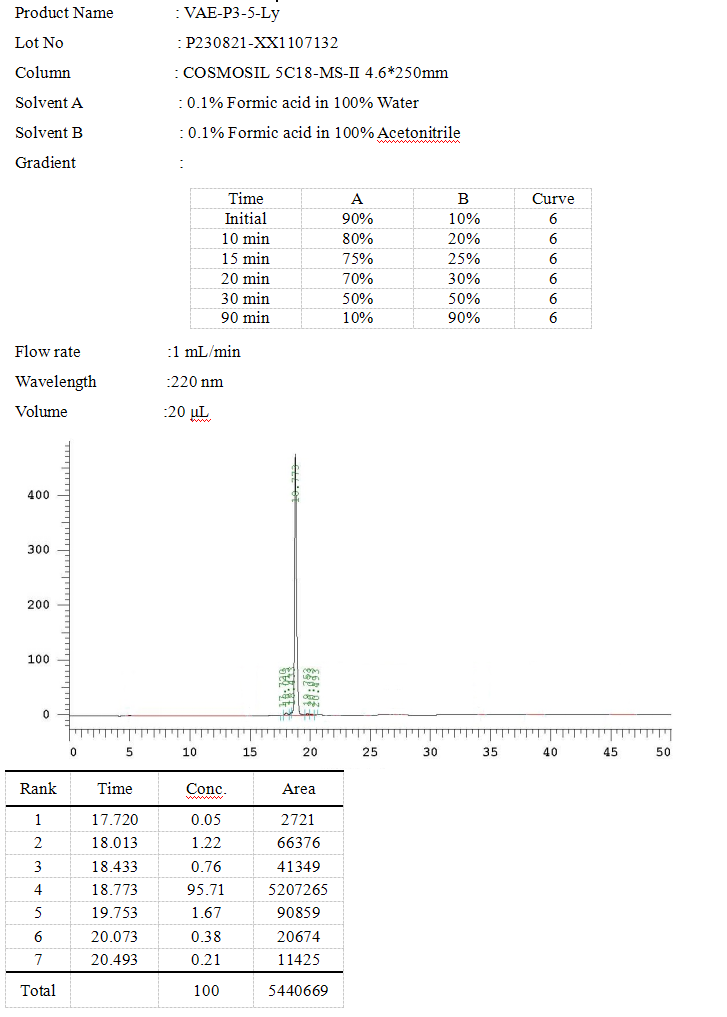
VAE-P3-5-Ly:

**Supplement S11** Stucture of synthetic staple peptides and linear peptides (LC-MS)

GAN-P2-2:


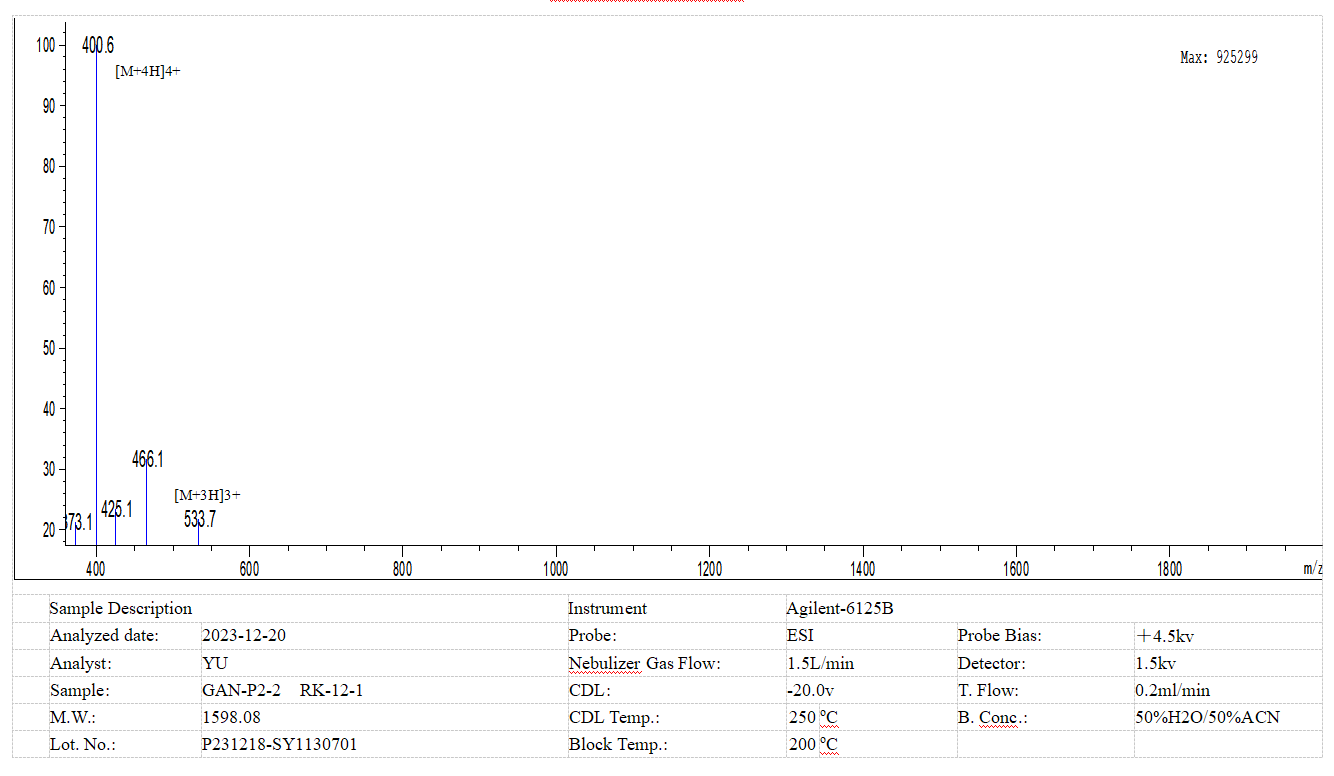


GAN-P2-2-Ly:


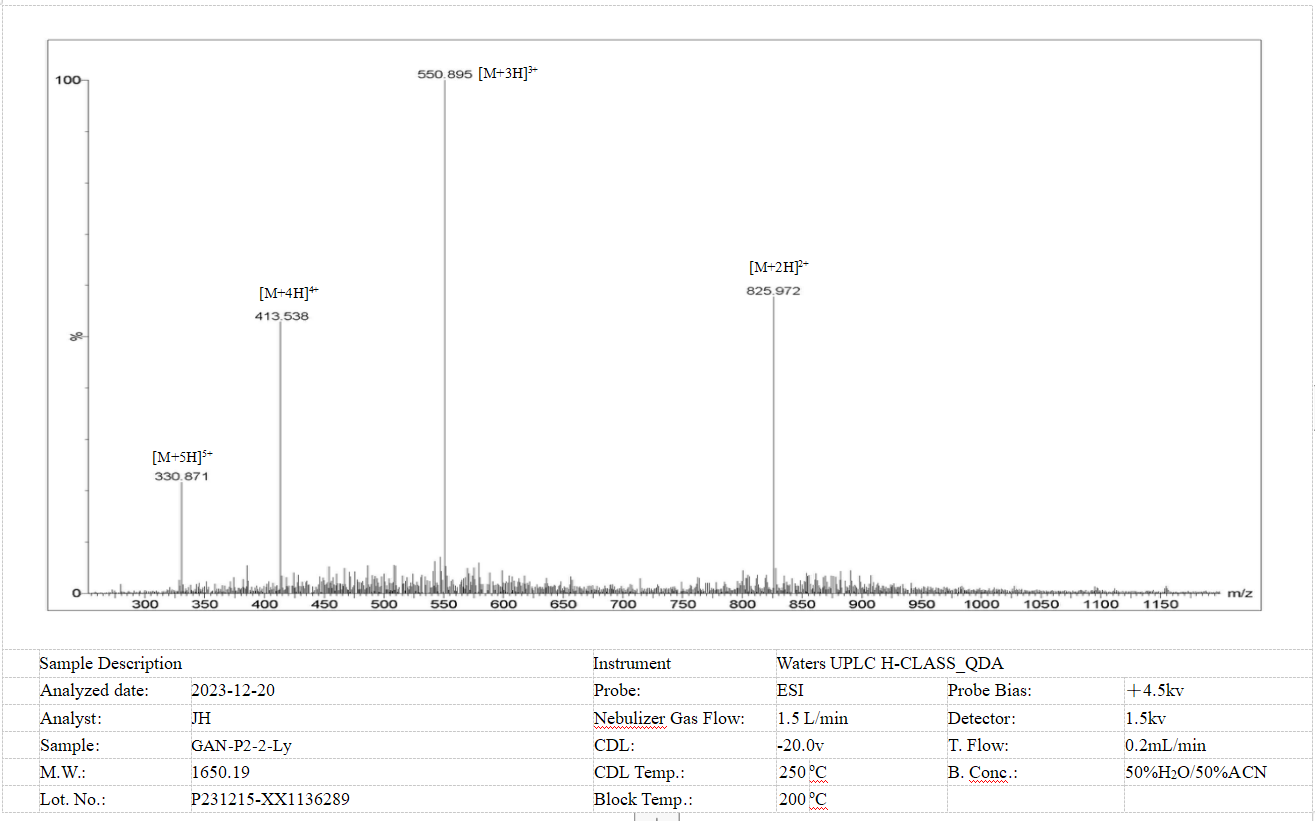


GAN-P2-3:


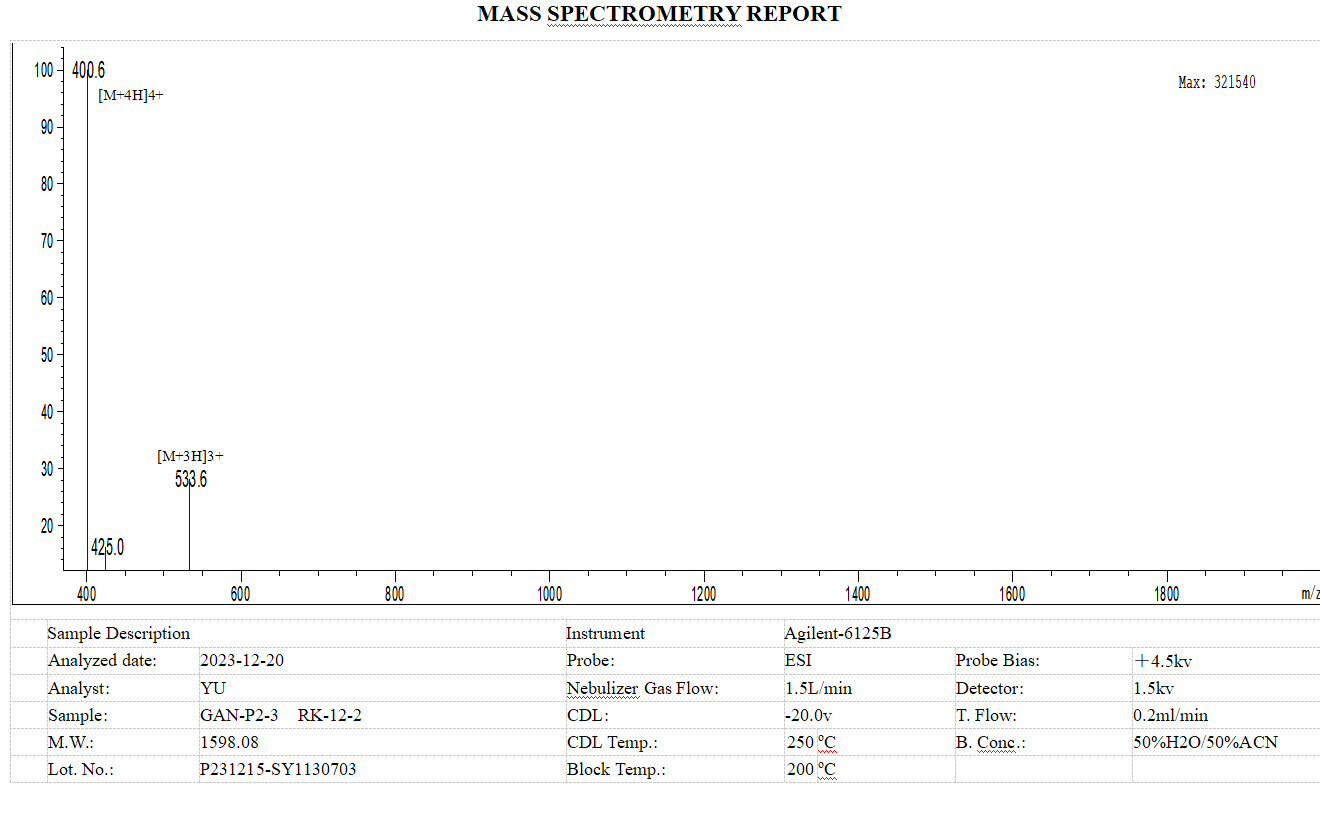


GAN-P2-3-Ly:


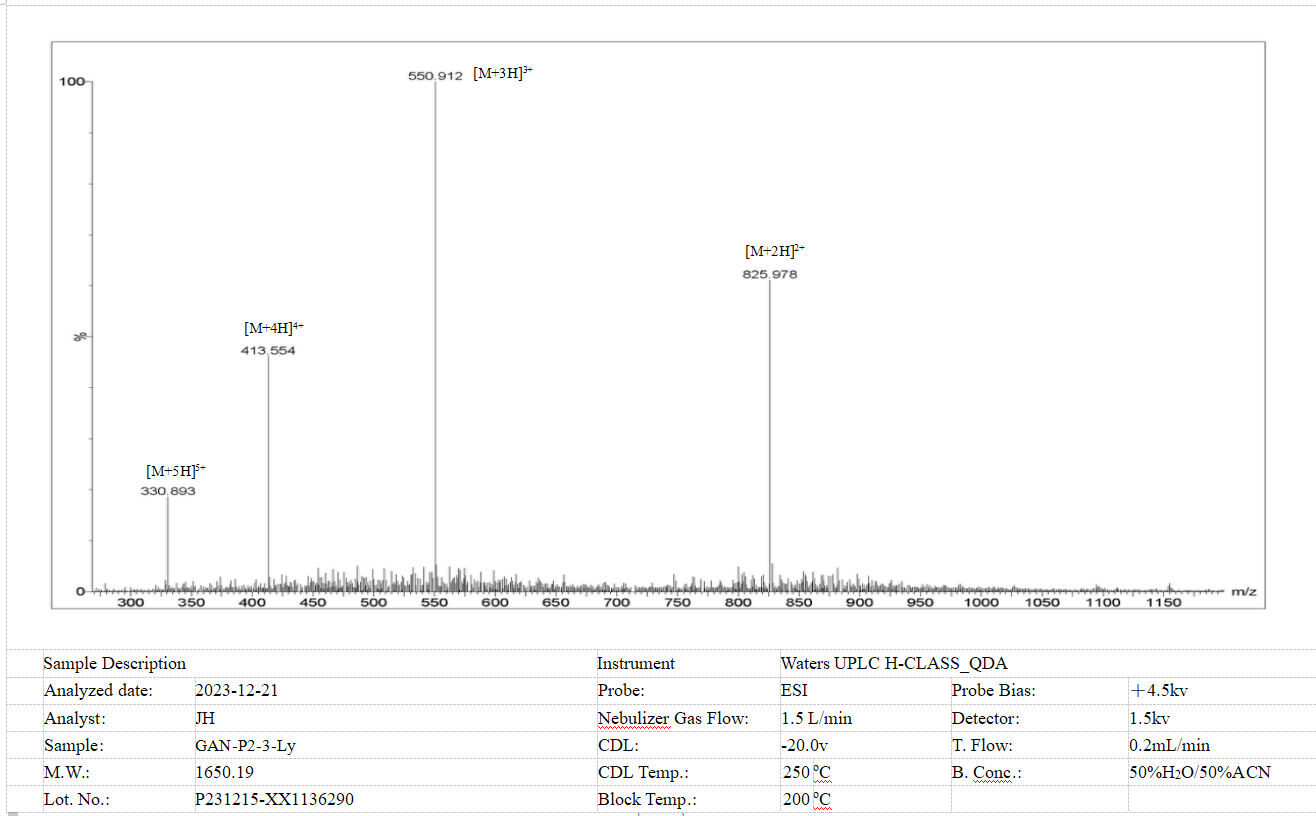


GAN-P2-4:


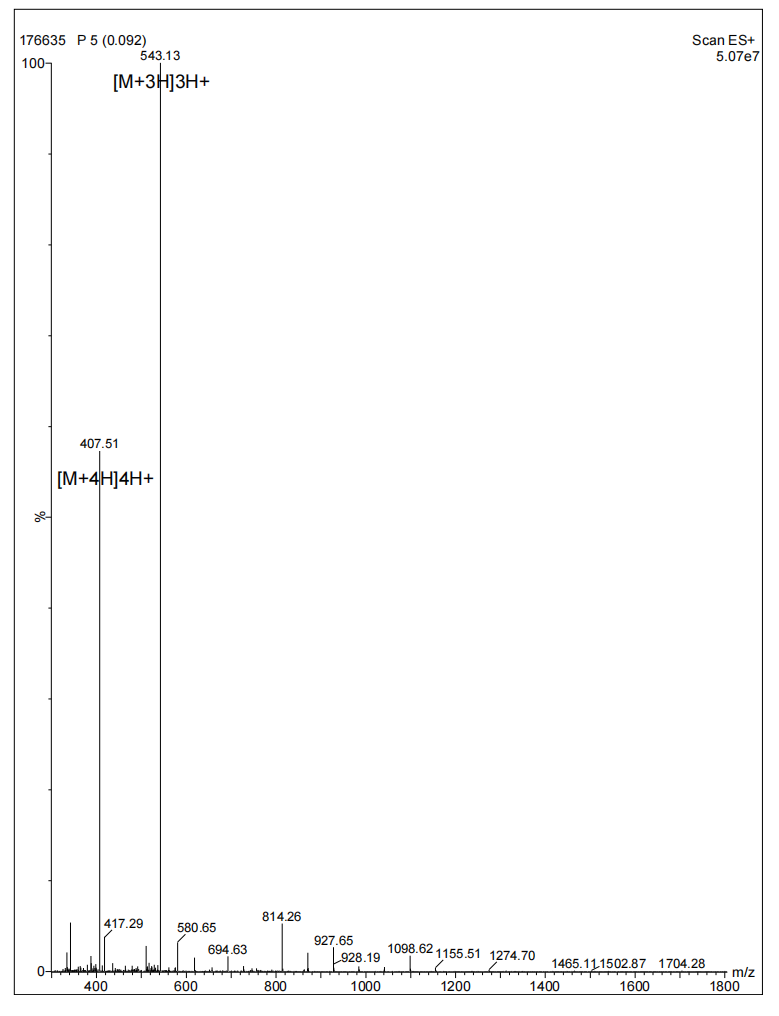


GAN-P2-4-Ly:


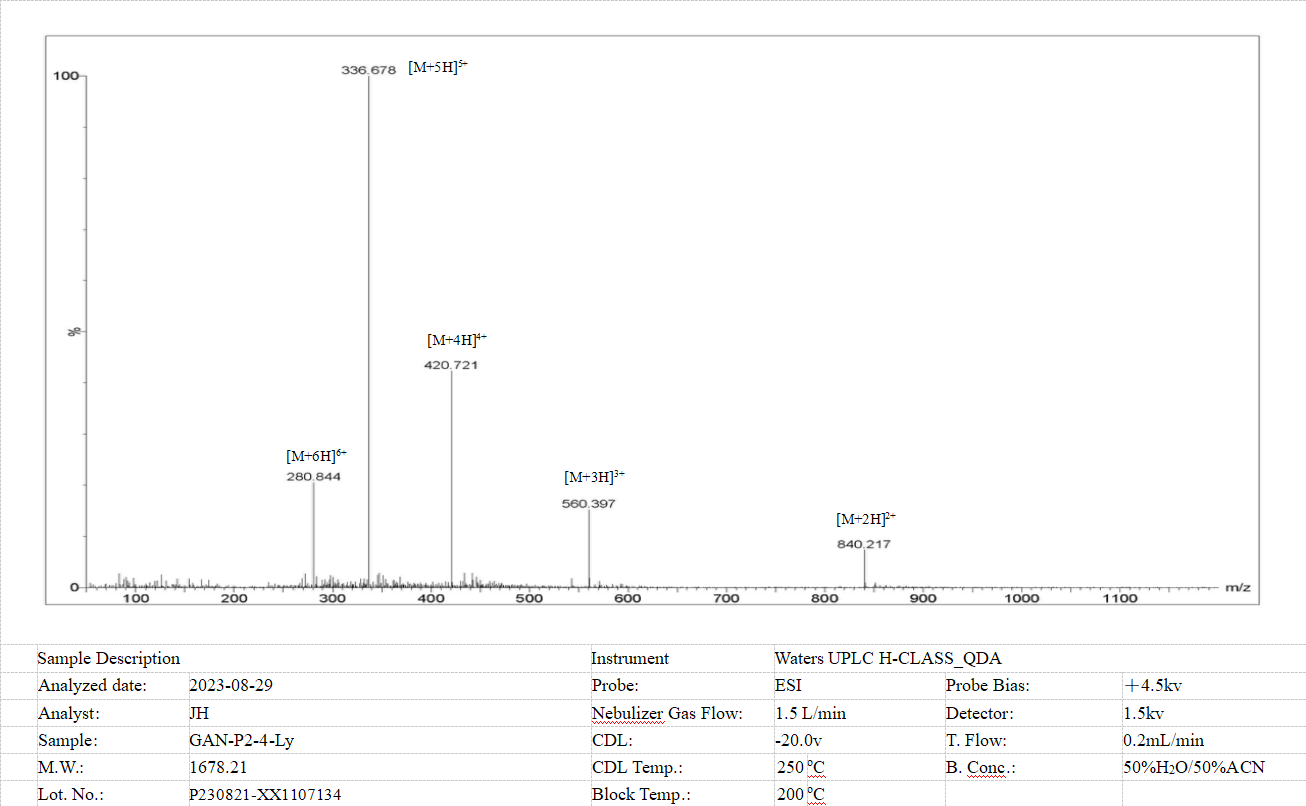


VAE-P3-3:


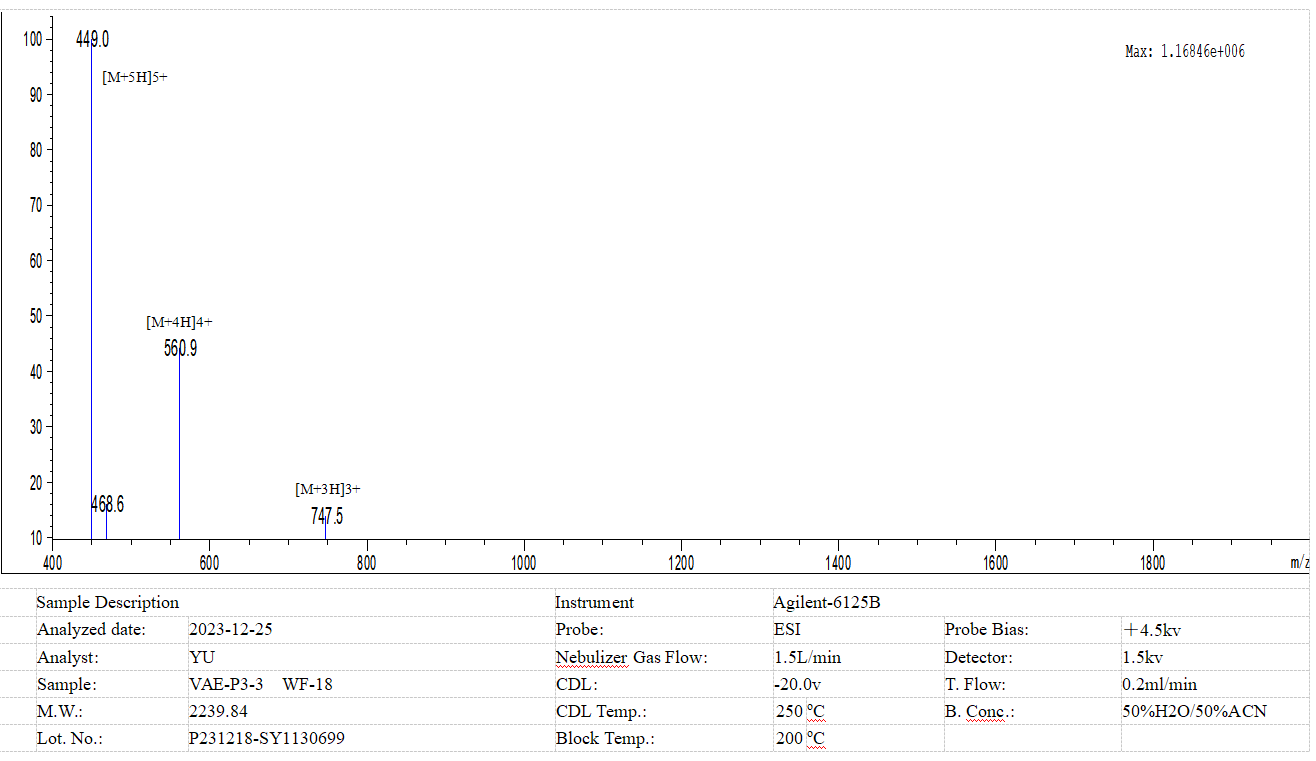


VAE-P3-3-Ly:


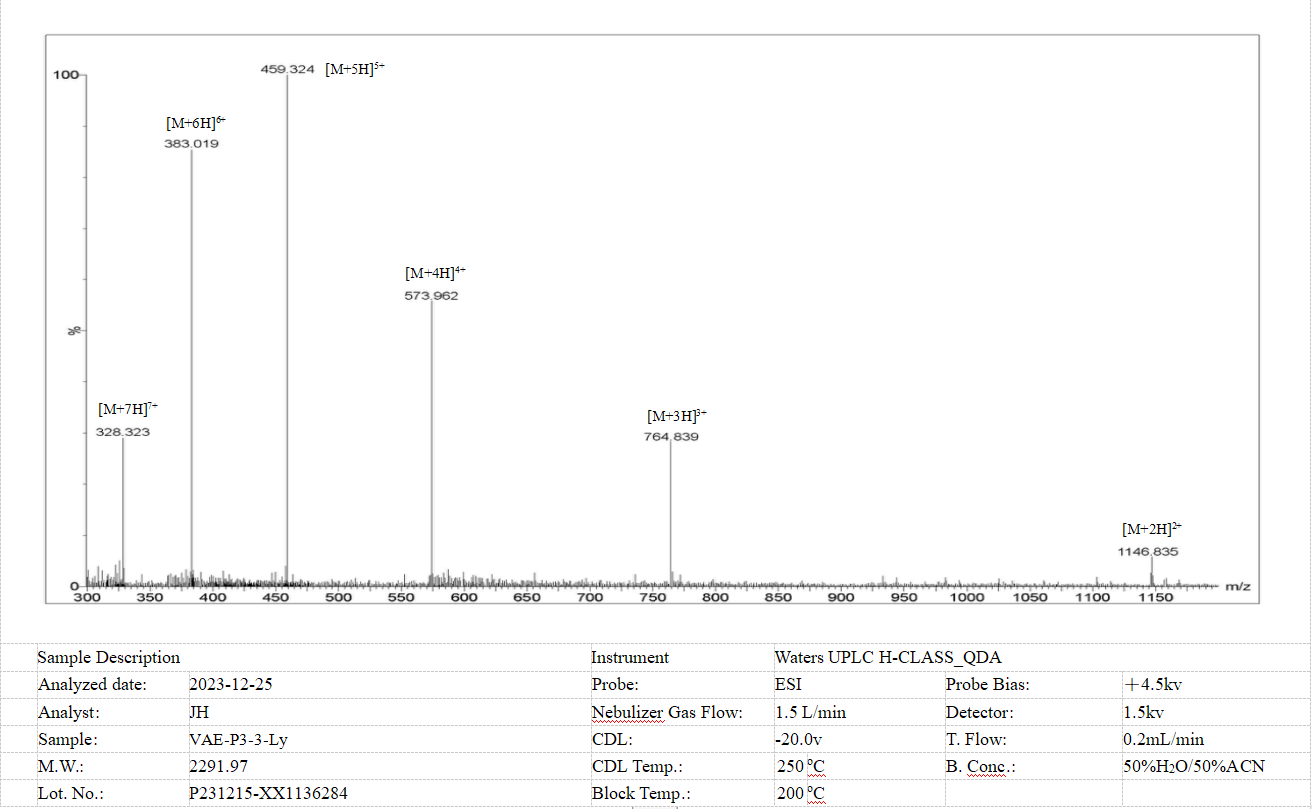


VAE-P3-5:


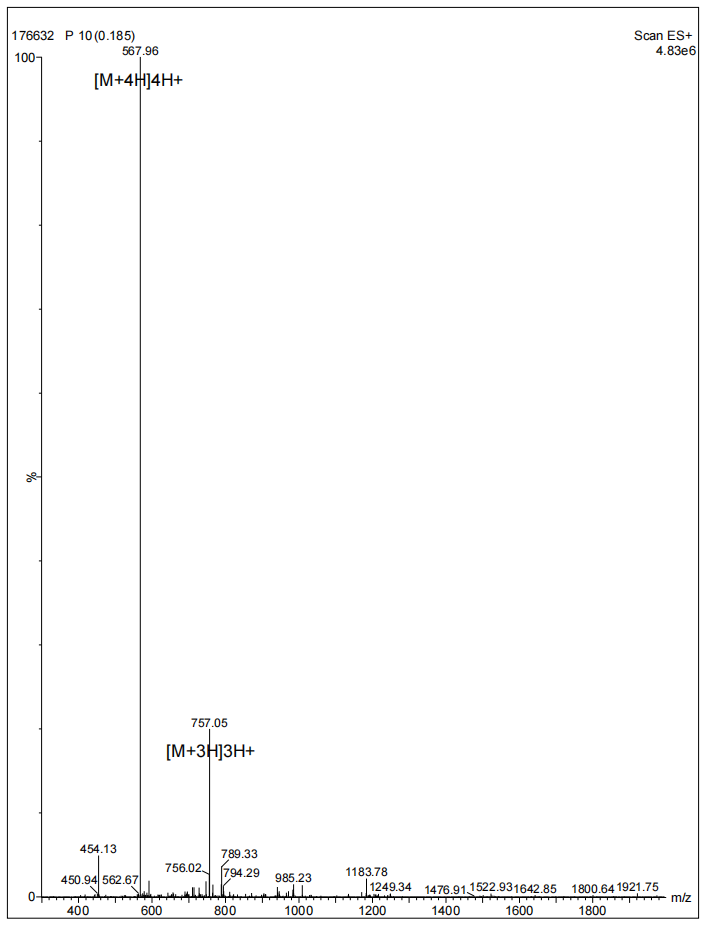


VAE-P3-5-Ly:


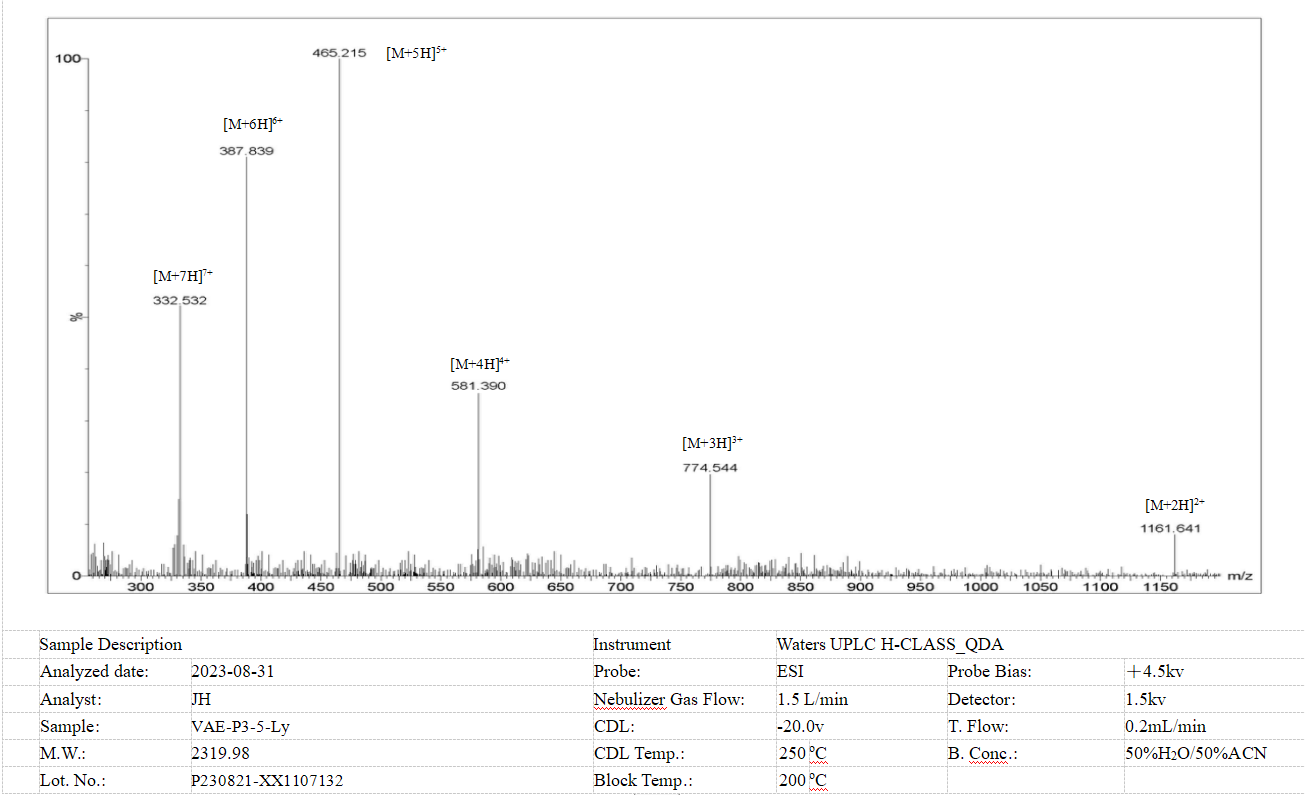


**Supplement S12** Predict result of experiment data set using ten models

| ID | Wet Exp. Result | Machine learning | | | | | | | Deep learning | | |
| --- | --- | --- | --- | --- | --- | --- | --- | --- | --- | --- | --- |
|  |  | DT | KNN | LGBM | NB | RF | SVM | XGB | ANN | CNN | LSTM |
| Mag2(i+4)14Dc | Positive | (+^a^)/T | (+)/T | (-^b^)/F | (+)/T | (+)/T | (+)/T | (-)/F | (+)/T | (+)/T | (+)/T |
| Mag2(i+4)17Dc | Positive | (+)/T | (+)/T | (+)/T | (+)/T | (+)/T | (+)/T | (-)/F | (+)/T | (+)/T | (-)/F |
| Mag(i+4)0St | Positive | (+)/T | (+)/T | (+)/T | (+)/T | (+)/T | (+)/T | (-)/F | (+)/T | (+)/T | (+)/T |
| Mag(i+7)11St | Positive | (-)/F | (+)/T | (+)/T | (+)/T | (+)/T | (+)/T | (+)/T | (+)/T | (+)/T | (+)/T |
| VAE-P3-3-St | Positive | (+)/T | (+)/T | (+)/T | (+)/T | (+)/T | (+)/T | (+)/T | (+)/T | (+)/T | (+)/T |
| VAE-P3-5-St | Positive | (+)/T | (+)/T | (+)/T | (+)/T | (+)/T | (+)/T | (+)/T | (+)/T | (+)/T | (+)/T |
| GAN-P2-2-St | Positive | (-)/F | (+)/T | (+)/T | (-)/F | (+)/T | (-)/F | (+)/T | (-)/F | (+)/T | (+)/T |
| GAN-P2-3-St | Positive | (-)/F | (+)/T | (+)/T | (-)/F | (+)/T | (-)/F | (+)/T | (-)/F | (+)/T | (+)/T |
| GAN-P2-4-St | Positive | (-)/F | (+)/T | (+)/T | (-)/F | (+)/T | (+)/T | (+)/T | (-)/F | (+)/T | (+)/T |
| Mag2(i+4)15Dc | Negative | (+)/F | (+)/F | (-)/T | (+)/F | (+)/F | (+)/F | (-)/T | (+)/F | (+)/F | (-)/T |
| Accuracy |  | 0.5 | **0.9** | **0.9** | 0.6 | **0.9** | 0.7 | 0.7 | 0.6 | **0.9** | **0.9** |

a: predict a score higher than 0.5.

b: predict a score with lower than 0.5.

T: True

F: False

**Supplement S13**  CD spectra of peptides in PBS (linear peptides in blue, stapled peptides in green), (A)GAN-P2-2 vs. GAN-P2-2-Ly, (B)GAN-P2-3 vs. GAN-P2-3-Ly, (C)GAN-P2-4 vs. GAN-P2-4-Ly, (D)VAE-P3-3 vs. VAE-P3-3-Ly, (E)VAE-P3-5 vs. VAE-P3-5-Ly：

(A)


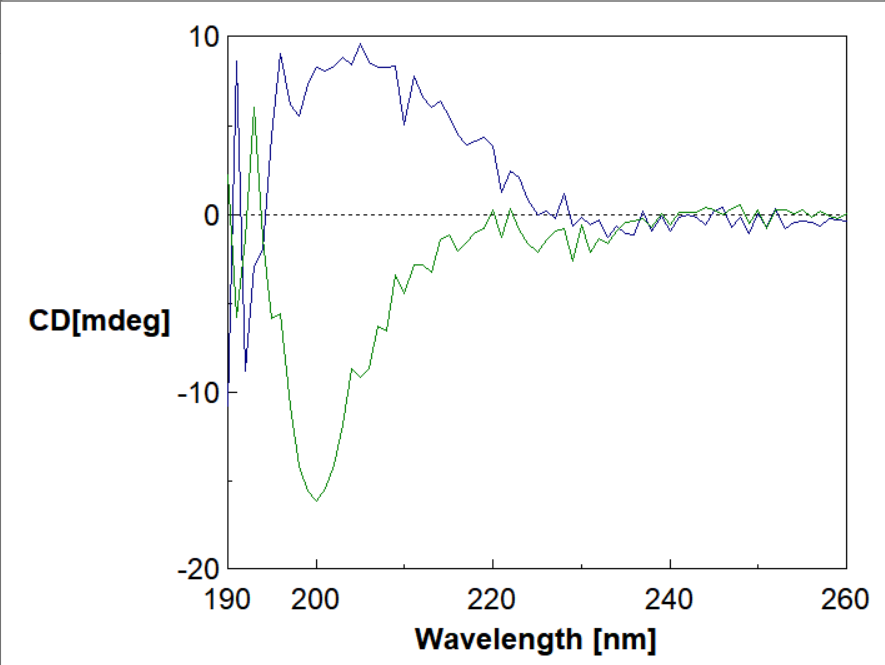


(B)


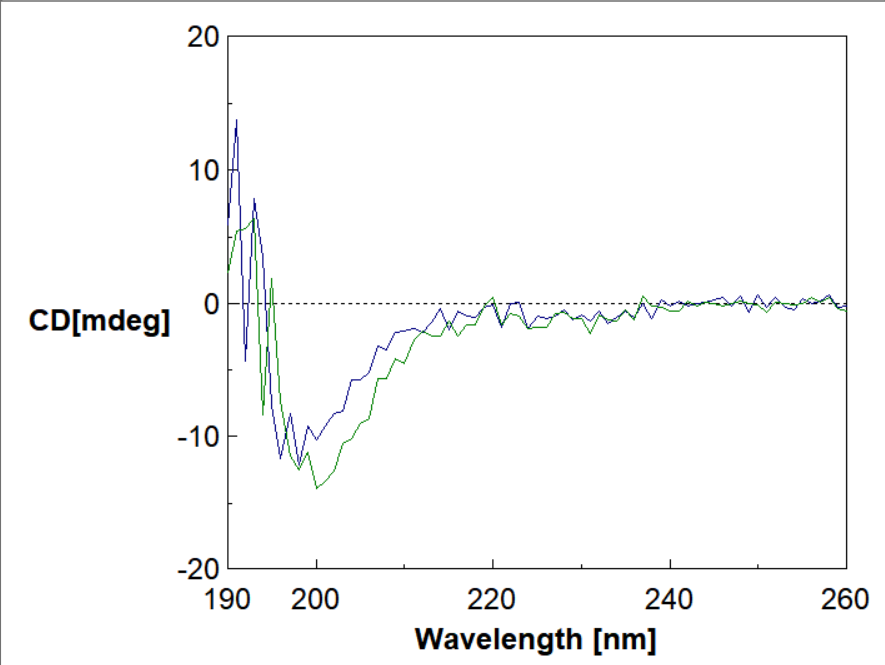


(C)


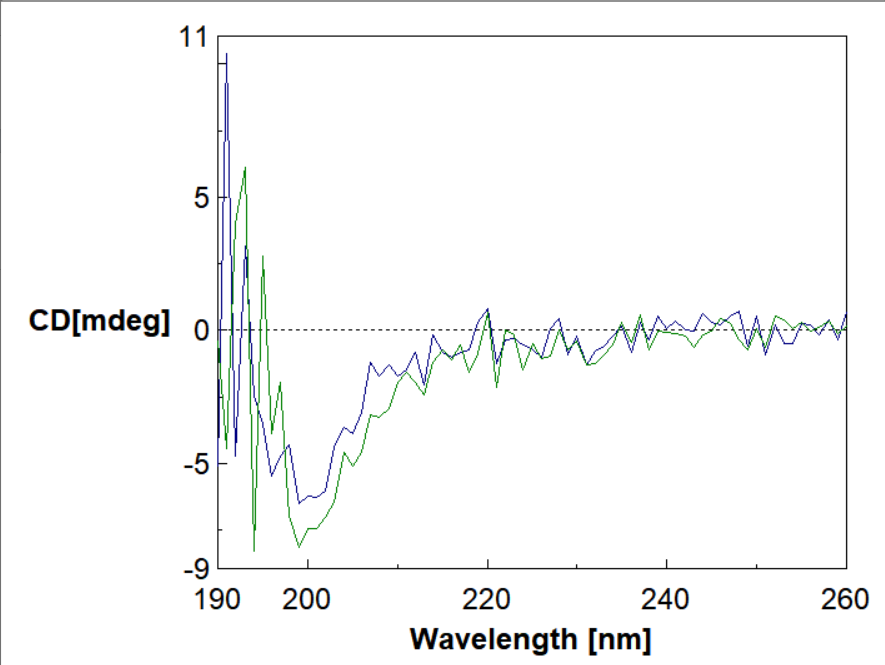


(D)


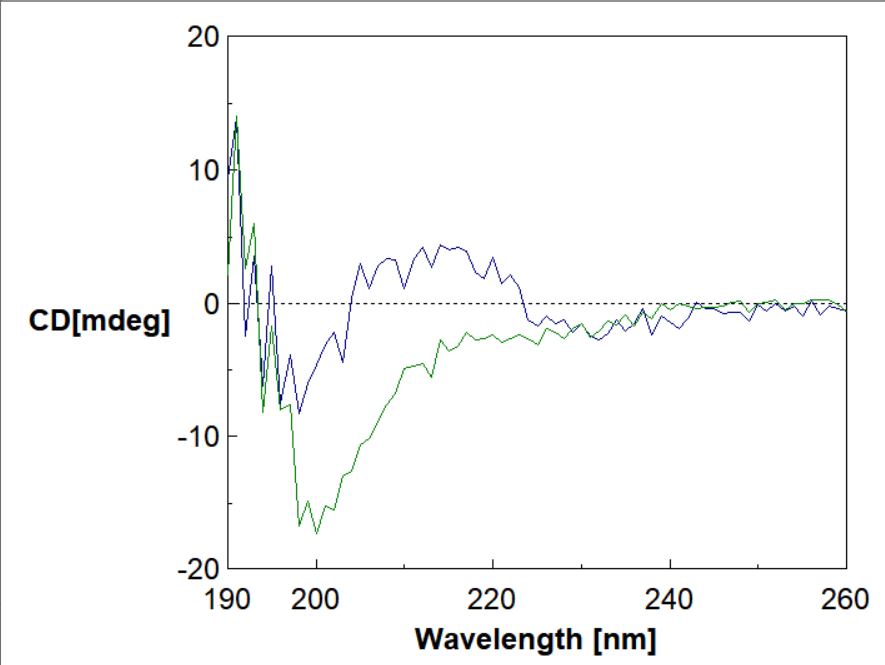


(E)


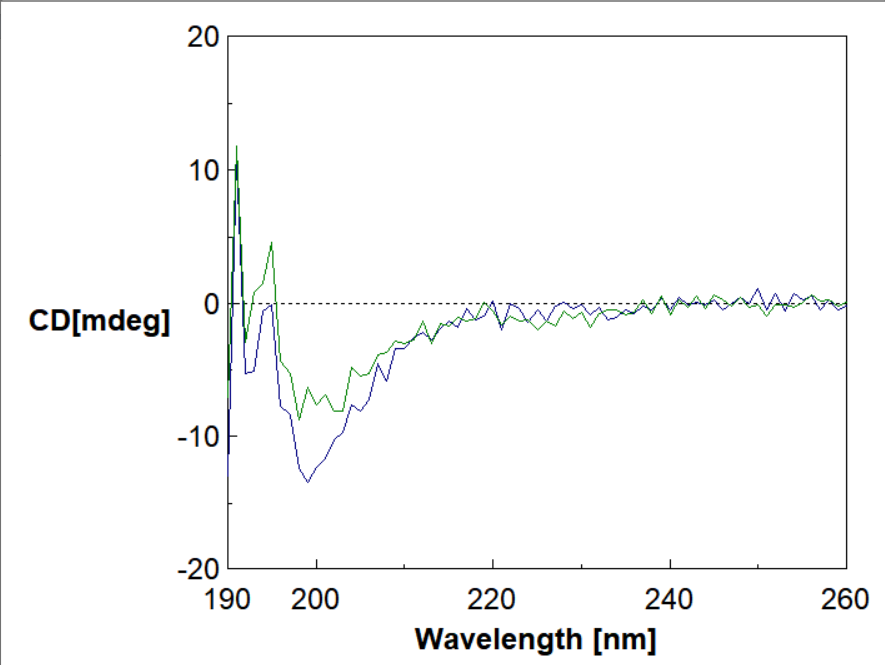

Supplement: Supplementary file 1 — Supporting Information S1. Supporting Information S2. Supporting Information S3. Supporting Information S5. Supporting Information S6. Supporting Information S7. Supporting Information S8. Supporting Information S9. Supporting Information S10. Supporting Information S11. Supporting Information S12. [file MBT2-18-e70121-s002.doc]
