# Supplementary figures and images for: Deep‐Learning‐Based Approaches for Rational Design of Stapled Peptides With High Antimicrobial Activity and Stability
Source: Microb Biotechnol. 2025 Mar 5;18(3):e70121. doi: 10.1111/1751-7915.70121 (PMC11881016; doi:10.1111/1751-7915.70121)

A

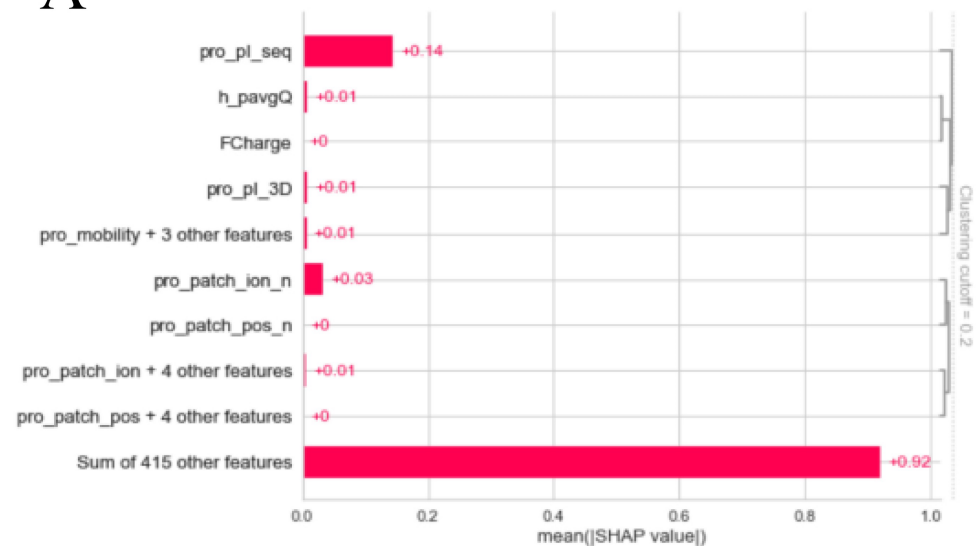

B

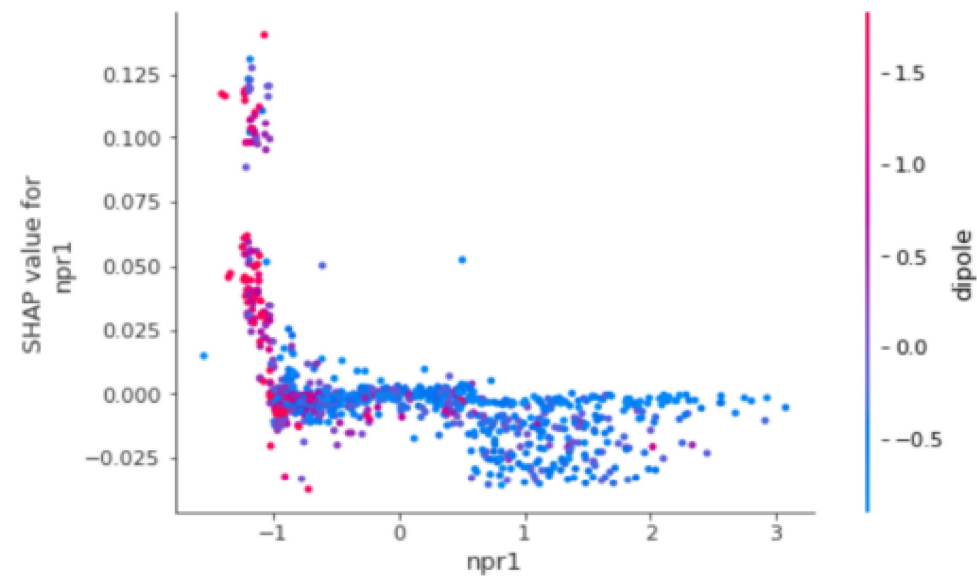

C

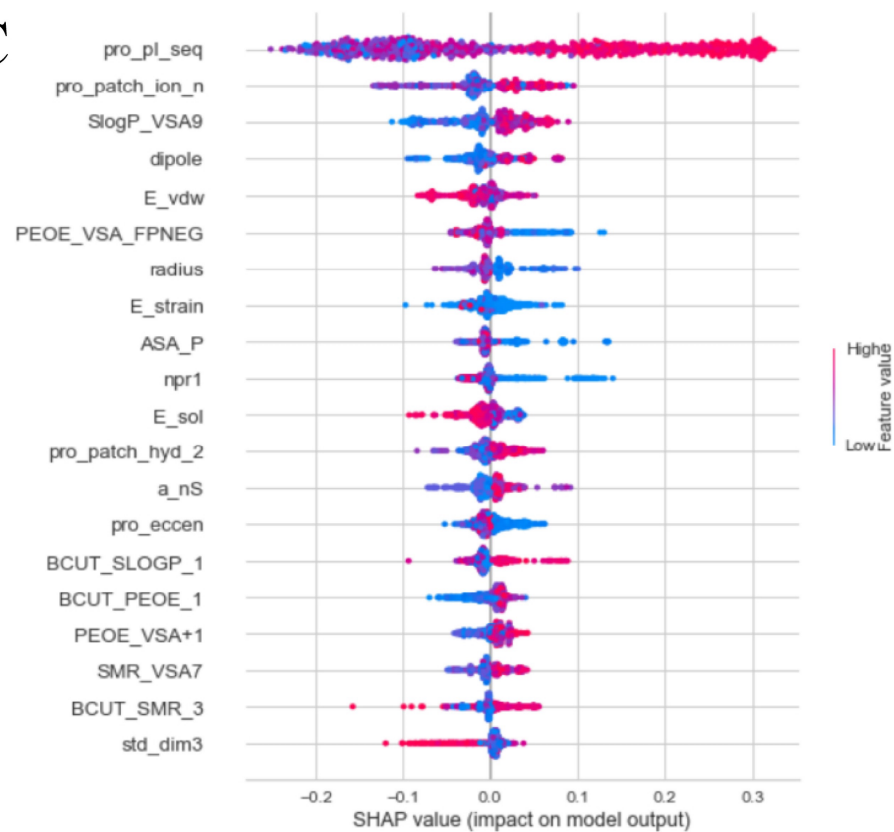

D

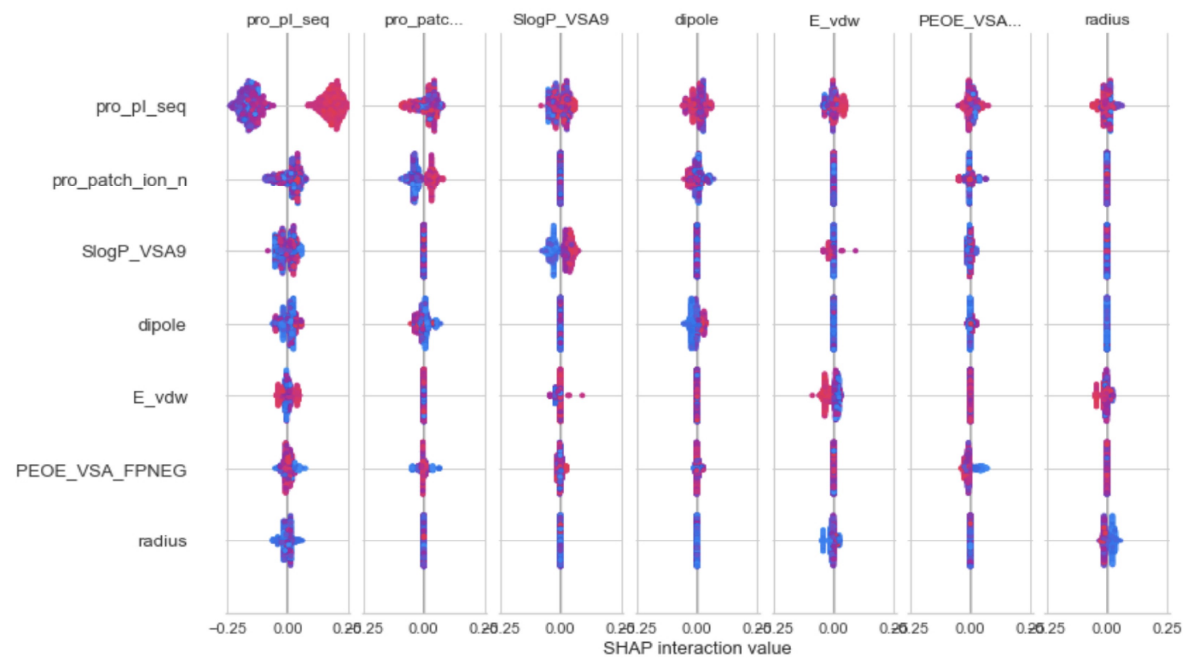

Supplement: Supplementary file 2 — Supporting Information S4. [file MBT2-18-e70121-s001.pdf]
